# Stem Cell and Exosome Therapy in Wound Healing: Traps, Paradoxes, and Tricks Transforming Paradigms

**DOI:** 10.3390/biomedicines13123030

**Published:** 2025-12-10

**Authors:** Gordana Velikic, Gordana Supic, Dusica L. Maric, Miljan Puletic, Marija D. Maric, Branko Barac, Dusan M. Maric

**Affiliations:** 1 Department for Research and Development, Clinic Orto MD-P.A.R.K.S. Hospital, 21000 Novi Sad, Serbia; ducamaric@gmail.com; 2 Hajim School of Engineering, University of Rochester, Rochester, NY 14627, USA; 3 Institute for Medical Research, Military Medical Academy, 11000 Belgrade, Serbia; gogasupic@gmail.com; 4 Medical Faculty of Military Medical Academy, University of Defense, 11000 Belgrade, Serbia; 5 Department of Anatomy, Faculty of Medicine, University of Novi Sad, 21000 Novi Sad, Serbia; 6 Faculty of Stomatology, Pancevo University Business Academy, 26000 Pancevo, Serbia; miljenko.puletic@gmail.com; 7 Faculty of Medicine, University of Novi Sad, 21000 Novi Sad, Serbia; marijamariconline@gmail.com; 8 Institute for Rheumatology, 11000 Belgrade, Serbia

**Keywords:** stem cell therapy, exosome therapy, wound healing, regenerative medicine, fibrosis and scarring, microenvironment, extracellular vesicles, bioelectric modulation, microbiome-exosome axis, paradigm shift

## Abstract

Cell therapies hold great promise for advancing wound healing; however, translating this promise into consistent clinical benefit has proven elusive. Numerous trials have failed to reproduce the robust outcomes suggested by preclinical studies, reflecting a landscape marked by hidden traps. These include the hostile wound microenvironment, the cytotoxicity of antimicrobial dressings, poor retention and engraftment, immune clearance, and the paradoxical risk of fibrosis and scarring. Across these challenges emerge paradoxes that redefine how traps are understood. The Scarring Paradox reveals that MSCs and EVs may either suppress or reinforce fibrosis, depending on the niche context. The Immune Double-Edged Sword captures the duality of clearance and regenerative modulation. These paradoxes illustrate that traps are not static obstacles but dynamic inflection points. Recognition of these paradoxes has inspired tricks: protective biomaterial carriers, preconditioning strategies, engineered exosomes, and combinatorial therapies with anti-fibrotic, neuromodulatory, or microbiome-targeted adjuncts. Case studies illustrate how classical traps manifest in clinical practice and how paradoxes guide innovation. Emerging adjuncts, ranging from herbal bioactives and bioelectric modulation to circadian synchronization and digital twins, point toward more unconventional but increasingly plausible frameworks for niche control. This perspective review demonstrates that the future of regenerative wound therapy depends not on avoiding traps but on reframing them through paradoxes and converting them into tricks. Stem cell and exosome therapy is thus moving beyond a linear “promise versus failure” narrative toward a systemic, context-aware, programmable approach in which paradoxes drive conceptual renewal and transformative paradigms in wound care.

## 1. Introduction

Wound healing is one of the most demanding areas in regenerative medicine, where biological complexity collides with urgent clinical need. Mesenchymal stem cells (MSCs) and their secreted extracellular vesicles (EVs), particularly exosomes, have been heralded as powerful tools to accelerate repair, reduce fibrosis, and restore tissue integrity [1,2]. Preclinical studies across animal models consistently demonstrate enhanced angiogenesis, modulation of inflammation, and improved epithelialization. These encouraging signals have driven a wave of clinical investigations in burns, diabetic ulcers, ischemic wounds, and surgical reconstruction.

However, the translational reality is sobering. Clinical trials have produced inconsistent outcomes, ranging from striking improvements to negligible or transient effects, with some reports of adverse events [3]. The gap between robust preclinical efficacy and variable clinical benefit underscores a persistent translational dilemma: the promise of MSC and exosome therapies is undeniable, but their performance in the human wound bed remains unpredictable [4,5,6].

This inconsistency is not simply the result of technical imperfections but reflects deeper traps embedded in wound biology and therapeutic practice. These traps include the hostile and dynamically shifting wound microenvironment, the cytotoxic side effects of standard dressings, the paradoxical roles of exosomes and stem cells that may either suppress or propagate fibrosis and senescence depending on context, and the fragile retention of transplanted cells and vesicles. Furthermore, an underrecognized trap lies in the influence of pathobiota: microbial, viral, and even fungal vesicles that silently interfere with regenerative signaling, thereby undermining the immune reprogramming that MSCs and EVs are designed to achieve. Importantly, recognizing these limitations has inspired a growing repertoire of innovative solutions, or “tricks,” that reframe obstacles into opportunities: from biomaterial carriers and exosome engineering to unconventional adjuncts such as bioelectric modulation or circadian synchronization.

This perspective review aims to map the landscape of traps, paradoxes, and tricks in stem cell- and exosome-based wound therapies. Rather than cataloging failures, this framework establishes new theoretical foundations that transform lessons of failure into predictive models for regenerative success. Novel integrative frameworks, including the Triad-Exosome Axis and the Multi-Axis Regeneration Map, are introduced to illustrate how apparent barriers can transform paradigms of wound care. By placing classical pitfalls alongside emerging and unconventional solutions, this review demonstrates that the future of regenerative wound healing lies not in bypassing traps, but in leveraging their paradoxical nature to generate tricks that ultimately transform paradigms.

### 1.1. Key Concepts in Traps, Paradoxes, and Tricks

To guide the reader, the following section defines several recurring concepts introduced in this review. These terms highlight how classical obstacles in wound healing can be reframed as drivers of conceptual renewal (Table 1).

This review deliberately adopts the metaphorical framework of “traps and tricks” to align with the Special Issue theme while providing a systematic reconceptualization of failures in regenerative medicine. “Traps” represent not merely obstacles, but predictable system vulnerabilities that can be decoded and addressed. “Tricks” are not simple workarounds, but strategic interventions that transform systemic liabilities into therapeutic opportunities. This framework moves beyond the traditional ‘challenges and solutions’ paradigm by emphasizing the dynamic, programmable nature of regenerative systems, where apparent barriers become entry points for intervention once their underlying logic is understood. To systematically illustrate this paradigm, each major section concludes with a “Trap-Trick Capsule” that synthesizes the key obstacles and their corresponding strategic solutions, building toward the integrative frameworks presented in the synthesis. When specific paradoxes govern a section’s traps and tricks, they are highlighted at the top of the capsule to illuminate the underlying conceptual logic. For clarity and ease of navigation, all capsules are compiled in the Supplementary Atlas of Trap–Trick Capsules (Supplement S1 with Supplementary Tables S1–S21), which provides a concise overview of the main paradoxes and their translational solutions discussed throughout the manuscript.

**Figure 1:**
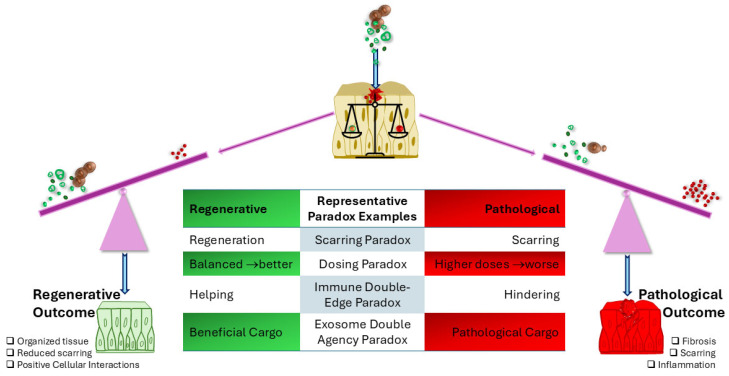
The Paradox Landscape in Regenerative Wound Therapy (Representative Examples). Four representative paradoxes are shown; additional paradoxes, including Vector, Preconditioning, and Temporal paradoxes, are detailed in the text. The balance scale illustrates how identical MSC and EV (represented as green and golden-brown particles) therapies can yield opposing outcomes depending on the niche context. Green vesicles represent therapeutic EVs with regenerative cargo, while red vesicles represent pathological EVs (vexosomes) carrying pro-inflammatory or fibrotic signals. The central table presents key paradoxes that govern therapeutic fate: the Scarring Paradox (regenerative vs. fibrotic outcomes), the Dosing Paradox (higher doses leading to worse outcomes through washout and immune clearance), the Immune Double-Edged Sword (protective vs. destructive immune responses), and the Exosome Paradox (therapeutic vs. pathological cargo delivery). The tissue cross-sections show opposing outcomes: organized regeneration with reduced scarring (left) versus fibrosis and chronic inflammation (right). Therapeutic success depends on identifying and manipulating the factors that tip this balance toward regeneration: optimizing niche conditions, timing delivery with permissive windows, filtering vexosomes, and coordinating multi-axis interventions. This framework demonstrates that therapeutic “failure” often reflects predictable paradoxes rather than random inconsistency, providing the conceptual foundation for the multi-systemic approaches that follow.

To illustrate how these paradoxes manifest, Figure 1 presents representative examples of the dual outcomes possible with identical regenerative therapies, establishing the conceptual foundation for the systems-level frameworks that follow.

**Methodological Note.** This article is written as a perspective review, intended to integrate existing evidence with hypothesis-generating frameworks. It does not follow the structure of a systematic review. Several conceptual terms (e.g., “bioelectric reset,” “vexosomes,” “neuroimmune scarring”) are introduced later in the manuscript as heuristic tools, proposed only after the foundational evidence is established to describe integrative mechanisms within the emerging regenerative paradigm.

### 1.2. Mechanistic and Translational Basis of MSC-Derived EVs

Before we dive deeper into traps, tricks, and paradoxes, it is essential to outline the biological foundations of the very therapies under discussion. MSCs and EVs form the core of regenerative approaches examined throughout this perspective review. MSCs exert their regenerative influence primarily through paracrine signaling, orchestrating repair rather than permanently engrafting into damaged tissue. Among their secreted factors, EVs, especially exosomes, serve as nanoscale messengers carrying regulatory microRNAs, cytokines, and growth factors that modulate inflammation, angiogenesis, and matrix remodeling.

Preclinical and early clinical evidence demonstrate that MSC-derived EVs accelerate closure of diabetic foot, venous leg, and burn wounds by suppressing inflammation (via miR-146a and Indoleamine 2,3-dioxygenase (IDO) signaling), promoting neovascularization (VEGF, PDGF), and stabilizing fibroblast-keratinocyte cross-talk. Innovative delivery strategies, including hydrogels, microneedle patches, and sprayable EV films, aim to extend EV residence time and preserve cargo bioactivity.

Despite this promise, challenges persist, including heterogeneity across cell sources, EV isolation methods, and potency assays, which complicate reproducibility and regulatory validation. Harmonized standards such as the ISEV 2023 minimal information framework and adherence to Good Manufacturing Practice (GMP) remain prerequisites for clinical translation.

**Stem Cells vs. Exosomes in Clinical Translation.** From a translational perspective, MSCs and their exosomes represent two stages of the same regenerative continuum rather than competing entities. MSCs retain broader biological plasticity and can dynamically respond to cues within complex wound niches; however, their clinical use is limited by immune clearance, poor engraftment, donor variability, and high manufacturing costs. Exosomes, as cell-free derivatives of MSCs, capture the therapeutic essence of stem cell signaling, delivering microRNAs, cytokines, and proteins that recapitulate paracrine effects, while minimizing the risks of tumorigenicity or ectopic differentiation. Their stability, scalability, and compatibility with off-the-shelf manufacturing make them particularly attractive for chronic and superficial wounds, where repeated, standardized applications are required. Nevertheless, MSCs remain indispensable in extensive or deep tissue injuries, where cell–cell interactions and niche remodeling exceed the capacity of vesicle-only therapies. Thus, while both modalities are complementary, exosomes currently hold greater near-term potential for broad clinical translation, whereas stem cell therapies continue to inform mechanistic understanding and serve as biofactories for next-generation vesicular products. A concise comparison of the translational features of stem cell- and exosome-based therapies is summarized in Supplementary Table S22.

**Manufacturing Stability and Quality as a Hidden Trap.** Beyond biological unpredictability, a critical barrier to clinical translation lies in the difficulty of producing stable, high-quality MSC or exosome products on a large scale. Variability in donor material, culture conditions, and isolation methods leads to inconsistency in potency and composition, creating what may be termed a manufacturing trap in regenerative medicine. Both stem cells and extracellular vesicles face stability constraints—cells through cryopreservation and transport viability, and vesicles through freeze–thaw degradation and cargo loss, limiting reproducibility across clinical sites. Converting this obstacle into a ‘trick’ requires standardized bioreactor systems, automated purification pipelines, and formulation technologies that ensure long-term stability and potency. Emerging lyophilized or polymer-stabilized EV formulations exemplify how manufacturing complexity can evolve from a translational liability into a design frontier for robust, on-demand regenerative therapeutics.

## 2. Classical Traps in Regenerative Wound Therapy

Despite the promise of stem cell and exosome therapies, their performance in the clinical wound setting has been hindered by a series of persistent and well-documented traps. These obstacles are not isolated but interwoven, reflecting the complex and often hostile biology of chronic or severe wounds. Preclinical studies in controlled environments frequently underestimate these challenges, which, in part, explains why clinical translation has been inconsistent. For transparency, Supplementary Table S23 summarizes the current regulatory and safety status of modalities mentioned in this perspective review, highlighting that many remain investigational outside controlled trials.

Several classical traps stand out. The wound microenvironment is characterized by oxidative stress, proteolytic activity, and disrupted bioelectric and metabolic gradients, all of which compromise the survival and function of transplanted cells or vesicles. Standard infection-control practices, such as silver- or iodine-based dressings, reduce microbial burden but simultaneously exert cytotoxic effects on MSCs and destabilize EVs. Poor retention and engraftment remain another major bottleneck, as transplanted products are often washed away, sequestered in dressings, or rapidly cleared by the immune system. Even when delivered successfully, stem cells and exosomes may not uniformly promote regeneration; in specific contexts, they can inadvertently favor fibrosis, hypertrophic scarring, or tumorigenesis through immune suppression and angiogenic signaling.

This section examines these classical traps in detail, highlighting the mechanisms underlying therapeutic failure and setting the stage for the corresponding tricks that have begun to emerge. By understanding how hostile wound biology undermines regenerative payloads, it becomes clear why past trials faltered, and how these very obstacles—often manifesting as paradoxes—may hold the key to reframing the paradigms of wound care.

### 2.1. Microenvironmental Hostility

The wound microenvironment is the stage and the gatekeeper of regenerative success [7]. For stem cell and exosome therapies, it represents the most immediate and formidable trap. Unlike controlled preclinical models, human wounds present a hostile and dynamically shifting landscape characterized by acidic pH, elevated lactate levels, proteolytic degradation, reactive oxygen species (ROS), and disturbances in ion flux [8]. These factors converge to compromise the survival, homing, and functional capacity of transplanted MSCs and EVs [9].

A defining but underappreciated element of this hostility is the disruption of endogenous bioelectric fields [10]. In intact epithelia, transepithelial potentials create directed electric fields of approximately 40–200 mV/mm, generated by ion fluxes across polarized layers. These fields act as guidance cues for keratinocytes, fibroblasts, and endothelial cells, orchestrating electrotaxis and coordinated migration during repair [11,12]. After injury, the breakdown of epithelial integrity leads to electrical disorganization: currents leak, polarity is lost, and cells receive incoherent directional cues. Instead of a sustained, vectorial signal that drives closure, the wound becomes electrically noisy, amplifying chaotic migration and delayed re-epithelialization [13]. Similarly, imbalances in sodium, potassium, and calcium flux further destabilize cell signaling and vesicle uptake, eroding the capacity of MSCs and exosomes to integrate with host tissue. For transplanted MSCs and EVs, this loss of polarity compounds their vulnerability, as regenerative cues are diluted in an environment lacking coherent spatial instruction [14,15]. In parallel, persistent oxidative stress generates an environment where therapeutic products are rapidly inactivated: cell membranes undergo lipid peroxidation, EV cargo is degraded, and mitochondrial damage in transplanted MSCs accelerates apoptosis. In this sense, electrical field collapse constitutes a “hidden trap” that is not immediately visible but profoundly influences the undermining of regenerative control.

Yet these same features, when understood, suggest strategic points of intervention that convert traps into therapeutic opportunities. Oxygen therapy, including normobaric or hyperbaric approaches, can reset local metabolism, thereby reducing lactate accumulation and stabilizing pH levels. Externally applied electrical stimulation seeks to restore lost wound currents, reestablishing directional cues for migration and electrotaxis [16,17,18]. Beyond these established approaches, several adjunctive modulators have emerged as promising compounds that support niche development. Antioxidants and redox-modulating therapies, such as N-acetylcysteine or curcumin derivatives, have been shown to mitigate oxidative stress and enhance MSC survival. N-acetylcysteine, a well-characterized antioxidant, directly counteracts ROS-mediated damage to MSC membranes and preserves EV cargo integrity [19]. Clinical studies demonstrate its capacity to reduce oxidative stress markers in chronic wounds while maintaining biocompatibility with regenerative cells. Similarly, curcumin derivatives offer dual benefits: their anti-inflammatory properties suppress excessive cytokine release that can overwhelm transplanted MSCs, while their antioxidant effects provide additional protection against lipid peroxidation and mitochondrial dysfunction [20,21].

Finally, biomaterial carriers such as hydrogels and extracellular matrix (ECM) scaffolds provide protective niches that buffer transplanted cells and EVs against immediate hostile exposure, prolonging their retention and paracrine activity [22].

Microenvironmental hostility illustrates that the wound niche is not a passive recipient but an active determinant of therapeutic outcomes (Supplement S1—Supplementary Table S1). Recognizing its traps and leveraging its vulnerabilities through redox modulation, bioelectric reprogramming, and niche engineering offers a roadmap for converting environmental adversity into regenerative opportunity through strategies such as the ‘bioelectric reset’ explored in Section 4.

### 2.2. Infection Control vs. Cytotoxicity

Infection management has always been a non-negotiable priority in wound care, as unchecked microbial growth not only delays closure but also poses systemic risks. Antimicrobial dressings, particularly those impregnated with silver or iodine, are among the most effective tools for controlling bacterial burden and biofilm formation [23,24]. However, this clinical necessity introduces a profound trap for regenerative therapies: the very agents that suppress pathogens also impair the survival and function of MSCs and EVs. Silver destabilizes cellular membranes and vesicle cargo, while iodine suppresses the proliferation and migration of host and transplanted cells. Even when microbial density is reduced, residual infection and biofilm persistence generate biochemical niches that are hostile to regenerative payloads [25].

Understanding this paradox is critical. The trap is clear: antimicrobial agents safeguard against infection but sabotage MSC- and EV-based therapies. The trick is to develop staged, context-aware strategies: short-term antimicrobial use for bioburden control, followed by cell-friendly dressings or carriers that protect regenerative therapies from collateral toxicity (Table 2). The following subsections examine these dynamics in detail, beginning with silver, iodine, and the persistent challenge of biofilm.

#### 2.2.1. Silver, Iodine, and Residual Infection/Biofilm

Infection control remains a cornerstone of wound management, yet the very strategies that suppress microbial growth often undermine regenerative interventions. Silver-based dressings exemplify this paradox. Silver sulfadiazine and silver-impregnated materials are widely used for their broad-spectrum antimicrobial activity. Still, they exert collateral cytotoxic effects on keratinocytes, fibroblasts, endothelial cells, and, critically, transplanted MSCs and EVs. Silver destabilizes vesicle membranes, disrupts mitochondrial respiration, and generates secondary oxidative stress, effectively neutralizing the regenerative payload [26].

Iodine-based preparations present a similar dilemma. While povidone-iodine and cadexomer iodine can reduce bacterial burden and biofilm density, they impair cell proliferation and migration at therapeutic concentrations [27,28,29,30]. Formulation differences are clinically relevant: cadexomer iodine provides controlled, sustained iodine release with lower transient cytotoxicity, whereas povidone-iodine delivers a rapid bolus effect, increasing immediate exposure and cell toxicity. For autologous or allogeneic cell therapies, this translates into poor engraftment and limited paracrine activity.

Regionally used antiseptics, such as brilliant green (zelyonka), a cationic triphenylmethane dye used for silk coloring, illustrate the same paradox [31,32,33,34]. Despite its historical popularity as a topical disinfectant, brilliant green is highly cytotoxic to keratinocytes, fibroblasts, and stem cells, reinforcing that antiseptic strategies that suppress microbes can simultaneously destabilize MSCs and EVs [35]. While diluted brilliant green has been historically observed to reduce bleeding, inflammation, and swelling, its mechanism of action—membrane disruption and oxidative stress—also makes it cytotoxic to keratinocytes, fibroblasts, MSCs, and EVs. Thus, the very properties that produce short-term symptomatic improvement may undermine long-term regenerative integration, illustrating the Infection-Control Paradox once again [36].

Even when antimicrobial dressings succeed in reducing the microbial load, residual infections and biofilm persistence remain significant traps. Biofilms create hypoxic, acidic niches rich in proteases, which degrade exosomal proteins and RNA cargo while shielding bacteria from immune clearance [37]. Moreover, bacterial EVs can mimic host exosomes, hijacking communication pathways and propagating pro-inflammatory or immunosuppressive signals that further destabilize the healing process [38,39].

The trick does not lie in abandoning antimicrobial dressings, but in sequencing and contextualizing their use. Silver and iodine may be indispensable during the acute infection phase, yet prolonged application erodes the very regenerative responses that cell and vesicle therapies aim to restore. Strategies include limiting the duration of cytotoxic dressings, employing non-adherent barriers that physically separate therapeutic cells from silver surfaces, and transitioning to biocompatible scaffolds or hydrogels once microbial control is achieved. Adjunctive therapies, such as quorum-sensing inhibitors, bacteriophages, or tailored antibiotics, may further reduce biofilm resilience, enabling a safer regenerative window for MSC or exosome therapies [40,41,42,43]. Complementing these targeted antimicrobial strategies, biocompatible alternatives offer infection control without MSC/EV toxicity. Medical-grade honey preparations, now FDA-approved for wound care, provide broad-spectrum antimicrobial activity through osmotic effects, low pH, and hydrogen peroxide generation, while preserving stem cell viability and function [44,45,46]. Hypochlorous acid (HOCl) solutions represent another promising approach. These naturally occurring antimicrobials eliminate pathogens through oxidative mechanisms, but unlike traditional antiseptics, they maintain compatibility with regenerative cells when used at appropriate concentrations [47]. These agents illustrate how infection control can be achieved without sacrificing the regenerative payload.

The infection-control paradox underscores a central theme: what protects the wound from microbes can simultaneously sabotage regeneration. It represents the most immediate trap clinicians face, before dosing or scarring even becomes relevant (Supplemental Table S2). Conceptually, it frames the tension between classical wound care priorities (sterility, antimicrobial safety) and new regenerative paradigms. Recognizing this balance is essential for translating stem cell and exosome therapies into consistent clinical benefit.

#### 2.2.2. Other Antibiotics and Antiseptics

Beyond silver and iodine, a wide range of antibiotics and antiseptic agents are routinely deployed in wound care. However, their interactions with stem cell- and exosome-based therapies remain poorly characterized. Many antiseptics, such as chlorhexidine, hydrogen peroxide, and polyhexanide, are effective against planktonic bacteria but exert collateral damage by disrupting lipid membranes and generating ROS [48,49,50]. These mechanisms, while valuable for microbial control, mirror the same pathways that destabilize transplanted MSCs and degrade EVs.

Systemic and topical antibiotics add another layer of complexity. Specific agents, including aminoglycosides and fluoroquinolones, are known to impair mitochondrial function or alter oxidative balance in mammalian cells. While these effects are generally tolerable in host tissue, they may significantly reduce the survival or differentiation potential of transplanted MSCs. Antibiotic pressure also reshapes the wound microbiome, which in turn influences vesicle signaling: depletion of beneficial commensals can eliminate supportive cross-talk, while resistant strains may secrete bacterial EVs that mimic or antagonize regenerative EVs.

The available evidence suggests a broader principle: agents designed to eliminate pathogens often undermine regenerative payloads through overlapping cytotoxic mechanisms. Unlike silver and iodine, which are well documented, the impact of other antibiotics and antiseptics on MSC and EV function remains underexplored. Addressing this gap is essential if cell- and vesicle-based therapies are to be integrated safely into clinical protocols where antimicrobials are indispensable.

To help readers navigate the variable strength of evidence across these commonly used wound-care modalities, we synthesized representative data from human, animal, and preclinical studies into an “evidence landscape”, Table 3. This summary does not replace systematic analysis, but rather orients the interpretation of the subsequent narrative. Evidence levels are graded as: A—human RCTs or meta-analyses; B—controlled animal or cohort studies; C—preclinical or in vitro; D—conceptual or hypothesis-generating.

These empirical anchors provide context for the next layer of paradoxes, where dosing dynamics, field effects, and cross-axis signaling redefine how regenerative cues operate beyond the biochemical domain.

### 2.3. Delivery and Retention Problems

A defining principle of stem cell biology is that regenerative capacity does not depend only on the intrinsic properties of the cell, but also on its niche—the specialized microenvironment that provides structural support, biochemical cues, and immunological context for survival and function [82]. In established tissues, niches regulate the balance between stem cell quiescence, activation, and differentiation, thereby ensuring both homeostatic maintenance and repair after injury. In solid organs, stem cell niches are inherently dynamic, but their activity is buffered within a protected architecture. Bone marrow, intestinal crypts, and hair follicle bulges, for example, cycle between quiescence and activation, responding predictably to homeostatic turnover or stress events such as infection and injury [83]. These niches are not static; they are finely regulated by ECM, vascular gradients, and immune cell interactions, which together provide a contained environment where stem cell activation is orchestrated and shielded from uncontrolled loss [84,85].

By contrast, the wound niche is assembled de novo after tissue injury and lacks this regulatory containment. It functions in an open system, continuously exposed to fluid exudation, microbial colonization, and mechanical shear. Microenvironmental cues fluctuate chaotically: ROS spike and subside, protease activity degrades ECM, pH oscillates between acidic and neutral, and bioelectric fields lose their polarity. Without the stabilizing “memory” of an established organ niche, transplanted cells or vesicles are rapidly lost, degraded, or inactivated—before exerting meaningful paracrine or regenerative effects [86]. Topically applied MSCs are easily washed away by wound fluid, phagocytosed, or lysed by neutrophil-derived enzymes. Intralesional injection improves initial deposition but often results in rapid cell death due to hypoxia, nutrient deprivation, and the absence of structural cues for anchorage. Exosomes and other vesicles, being nanoscale, are particularly vulnerable: they diffuse freely, are absorbed into wound dressings, or undergo endocytic clearance without reaching target cells. Even when MSCs or EVs are administered into a wound under optimal conditions, a significant barrier to efficacy is their poor retention and limited functional persistence. This distinction highlights why delivery and retention remain such critical traps in wound healing. In solid organs, regenerative therapies can leverage existing niche dynamics. In wounds, the absence of structural protection means that survival depends almost entirely on the development of artificial niches, such as hydrogels, ECM scaffolds, or bioengineered carriers, that recreate buffering functions and prolong therapeutic presence.

Another important but underappreciated limitation is the “dosing paradox.” Preclinical studies often employ very high doses of MSCs or EVs, assuming that greater numbers will overcome the hostile wound niche [3]. Paradoxically, large bolus doses can worsen therapeutic loss. Higher volumes increase convective washout, pushing cells or vesicles out of the wound and into the dressing [87]. Dense inocula compete for scarce oxygen and anchorage, creating metabolic stress and accelerating apoptosis [88,89,90]. Excessive paracrine signaling can also trigger receptor desensitization or negative feedback loops in host cells, blunting responsiveness to regenerative cues [88]. Finally, more transplanted cells can release more damage-associated molecular patterns (DAMPs) as they die, amplifying immune clearance and inflammation [91,92,93]. In this way, the very strategy of “more is better” becomes a trap, leading to less effective exposure of the wound to regenerative signals [94] (Supplemental Table S3).

A striking example of this paradox is seen in specific clinical and experimental protocols that involve bathing the wound or organ in a suspension of stem cells or stem cell-conditioned medium [95]. The rationale is that flooding the tissue surface ensures maximum exposure to regenerative cues. However, in practice, this approach mirrors the pitfalls of bolus overdosing. Bathing fluid is rapidly lost through drainage or absorption into dressings, resulting in convective washout of transplanted cells and vesicles [96,97]. The high concentration of paracrine factors delivered at once hypothetically risks receptor desensitization and negative feedback in host cells, while large numbers of unattached MSCs undergo stress and apoptosis in suspension, releasing DAMPs that trigger immune clearance [98]. Without structural support, exosomes and vesicles in the bathing medium are exposed immediately to ROS, proteases, and pH shifts, accelerating degradation [99]. Additionally, the transient nature of bathing solutions means therapeutic contact time is often insufficient to establish meaningful cellular interactions or allow proper cellular engraftment.

Nonetheless, bathing need not remain a trap if reframed as a niche-aware strategy. Embedding MSCs or EVs in hydrogels, ECM fragments, or bioadhesive coatings can convert a fleeting wash into a retentive surface application [100]. Similarly, fractionated irrigation: multiple small-volume exposures rather than a single flood, reduces metabolic shock and allows the host niche to adapt. Sequential bathing protocols that alternate between therapeutic solutions and stabilization periods can enhance cellular adaptation and reduce washout losses. Supplementing the bath with protective agents such as antioxidants or pH-buffering systems and integrating it with bioelectric stimulation can further buffer the transplanted payload. Preconditioning the wound bed with mild debridement or controlled inflammation can also improve cellular receptivity to subsequent bathing treatments. In this way, stem cell bathing vividly illustrates the dosing paradox: as a raw bolus, it undermines regeneration, but with contextual redesign, it has the potential to serve as a supportive strategy.

The corresponding approach is fractionated, niche-aware dosing and delivery. Smaller, repeated doses allow the system to adapt gradually, reducing washout and avoiding receptor saturation (Table 4). Microvolume applications, such as sprayable fibrin films or microneedle patches, deliver concentrated cues without overwhelming the niche. Timing these applications to coincide with the natural phases of wound healing can further optimize therapeutic uptake. Encapsulation in protective carriers: hydrogels, ECM scaffolds, or 3D microgels—buffers metabolic stress, slows release, and shields against immune recognition. Preconditioning MSCs (e.g., hypoxia, cytokine priming) or stabilizing EVs with antioxidants further enhances survival. Real-time monitoring of wound biomarkers can guide dosing frequency and concentration adjustments. These strategies illustrate that success depends less on escalating dose than on smart delivery: fractionated, protected, and synchronized with the wound’s capacity to respond.

These limitations explain why the high doses reported in many preclinical models often fail to translate into sustained clinical benefit. Importantly, the failure is not merely quantitative but qualitative: transient exposure to regenerative signals is insufficient to override chronic inflammatory or fibrotic trajectories. Without strategies that anchor or repeatedly deliver MSCs/EVs, the therapeutic signal is drowned out by the hostile milieu.

Tricks to address this problem focus on creating niches that prolong retention and functional signaling. Biomaterial scaffolds, ranging from fibrin glue and collagen matrices to hyaluronic acid and hydrogel composites, provide physical protection and allow gradual release of regenerative cues. Spray systems, microneedle patches, and 3D-printed carriers have emerged as innovative platforms to enhance deposition and integration. Systemic approaches, such as exosome modification with targeting ligands, aim to increase homing and reduce off-target clearance. Collectively, these strategies suggest that successful translation does not depend on ever-higher doses, but on smarter delivery systems that stabilize regenerative therapies within the wound until healing trajectories are reprogrammed.

#### Pathological EVs as Hidden Traps

Not all EVs act as regenerative messengers. Emerging evidence highlights classes of pathological EVs that actively derail healing. Senescence-associated EVs (SA-EVs) propagate pro-inflammatory and pro-fibrotic signals, amplifying tissue aging and scar formation [101,102]. Microbial EVs, released by bacteria and fungi, can penetrate host cells and mimic host signaling, thereby sustaining chronic inflammation or inhibiting MSC-derived signals [103,104]. Fibrosis-associated EVs, derived from activated myofibroblasts, reinforce collagen cross-linking and ECM stiffening, perpetuating scarring loops [105,106]. Together, these vesicles constitute a double-edged sword: while therapeutic EVs can guide regeneration, pathological EVs may neutralize or even reverse their effects. The recognition of these hidden players reframes EV therapy not as a uniform good, but as a context-dependent gamble where filtering, neutralization, or engineering are essential counter-tricks [107].

Together, these vesicles illustrate that extracellular signaling is not inherently regenerative. To capture this broader category of deleterious vesicles, emphasizing their shared maladaptive signaling features, i.e., those that propagate inflammatory, fibrotic, or microbial distress signals, we refer to them here as “vexosomes.” This term denotes EVs whose cargo actively derails repair processes rather than supporting them. The concept is further developed in Section 6.3, where the vexosome framework is expanded to integrate cellular stress, microbiota, and immune cross-talk within the broader regenerative paradigm.

### 2.4. Fibrosis and Scarring Bias

Fibrosis is the pathological overproduction and deposition of ECM components—primarily collagen, driven by sustained myofibroblast activation [108]. It leads to tissue stiffening, altered architecture, and loss of function. Fibrosis is fundamentally a systemic problem, arising not only in wounds but also in organs such as the lungs, liver, kidneys, and heart, where it underlies chronic disease progression [109,110]. Scarring, by contrast, represents a localized form of fibrosis in the skin, where rapid deposition of ECM restores barrier integrity but sacrifices elasticity, adnexal structures, and full regenerative capacity [108]. While systemic fibrosis threatens organ function and survival, cutaneous scarring is functionally limiting, impairing mobility, cosmesis, and quality of life [111]. From an evolutionary perspective, scarring is advantageous: it prioritizes survival by closing wounds quickly. From a regenerative perspective, however, it is a significant obstacle, locking repair into a fibrotic rather than a regenerative trajectory.

One of the most persistent obstacles in wound healing is the intrinsic bias toward fibrosis and scarring. For the cell-based therapies, this bias represents a central trap: regenerative payloads introduced into a wound may be co-opted into fibrotic rather than regenerative trajectories.

At the molecular level, this bias is reinforced by transforming growth factor-β (TGF-β), connective tissue growth factor (CTGF), and YAP/TAZ-driven mechanotransduction, which promote myofibroblast activation and excessive ECM deposition [109,112]. Even MSCs and their exosomes, often promoted as anti-fibrotic, can display context-dependent duality [113,114]. In permissive environments, they suppress scar formation by tempering inflammation and reducing myofibroblast persistence. However, in dysregulated niches, they may inadvertently amplify fibrosis, secreting factors that drive fibroblast proliferation, collagen cross-linking, and the development of hypertrophic scarring [115]. This dual role exemplifies the Scarring Paradox: stem cell and exosome therapies can either attenuate or exacerbate fibrosis, depending on the state of the host niche (Supplemental Table S4, Table 5).

Clinically, this paradox is evident in burns and chronic wounds, where MSC or exosome therapies sometimes accelerate closure but yield rigid, hypertrophic scars with limited functional recovery. The trick lies in modulating the fibrotic switch rather than attempting to bypass it altogether. Several hypothetical and emerging strategies are listed below:

- Anti-fibrotic adjuvants such as quercetin, losartan, or verteporfin (YAP/TAZ inhibitor) reduce scarring signals when co-delivered with MSCs or EVs [116].
- Senolytic approaches eliminate senescent fibroblasts that act as chronic sources of fibrotic signaling [101].
- Preconditioning MSCs to bias their secretome toward anti-fibrotic factors (e.g., under hypoxic or anti-TGF-β environments [114]).
- Biophysical modulation through soft hydrogels or aligned nanofibers that guide tissue repair away from disorganized ECM deposition [117].

Ultimately, overcoming fibrosis requires acknowledging that the wound niche is “hardwired” for scarring. Therapies succeed when they convert scar-forming programs into regenerative programs, leveraging the paradox rather than ignoring it.

#### The Microbiota-Exosome Axis: Hidden Drivers of Fibrosis

The clinical community has long viewed the microbiota of skin and mucosa as background noise in wound healing [118,119,120]. However, mounting evidence suggests that dysbiotic microbial communities actively shape fibrotic outcomes. Microbes release EVs that carry proteins, metabolites, and nucleic acids, which can penetrate host cells and mimic host signaling. When this pathobiota hijacks the intrinsic exosomal dialog, it reinforces inflammation and drives maladaptive fibroblast activation.

In keloids and hypertrophic scars, microbial EVs appear to collaborate with fibroblast- and mast cell-derived EVs, amplifying collagen cross-linking, ECM stiffening, and contractile scarring [121,122,123]. This interaction creates a self-perpetuating loop: microbial EVs sustain inflammatory tone, while fibrotic EVs reinforce scar rigidity. Together, they establish a Microbiota-Exosome Axis that transforms wounds from regenerative attempts into pathological fibrosis.

Latent infections intensify this axis. Herpesviruses, papillomaviruses, and retroviruses can remain clinically silent yet alter host EV profiles, loading them with viral microRNAs or proteins that prime fibrosis [124,125,126,127,128]. When viral EVs intersect with bacterial or fungal EVs (e.g., *Candida* species), the result is a multi-microbial trap: cross-kingdom vesicle signaling that overwhelms regenerative MSC/EV cues [129,130]. Such hidden enemies undermine systemic processes, shifting the wound niche toward persistent inflammation, fibrosis, or even malignant transformation.

Tricks to counter this axis are emerging. Targeted antimicrobials, bacteriophages, or quorum-sensing inhibitors can disrupt pathogenic biofilms; probiotics and microbiome engineering can restore balanced microbial-host EV signaling; and engineered MSC/EV therapies may selectively neutralize or outcompete vexosomes. Recognizing the Microbiota-Exosome Axis reframes fibrosis not only as a host-driven bias, but as the outcome of a dynamic ecological battle between regenerative and pathological vesicles.

### 2.5. Immune Clearance and Tumorigenesis

The immune system represents a guardian and an adversary for stem cell and exosome therapies. On the one hand, innate and adaptive responses clear transplanted products, reducing retention and functional persistence. On the other hand, immune modulation is one of the mechanisms by which MSCs and EVs are expected to promote regeneration. This duality forms the Immune Double-Edged Sword: the same immune pathways that enable therapeutic benefit also accelerate therapeutic loss.

Clearance begins almost immediately after delivery. Neutrophils, macrophages, and complement proteins recognize transplanted MSCs as stressed or foreign, particularly when cryopreservation or culture stress induces expression of danger-associated molecular patterns (DAMPs) [131,132]. Phagocytosis, oxidative burst, and protease release then reduce viable cell numbers within hours. EVs are likewise opsonized and internalized by macrophages, with many vesicles trafficked to the liver, spleen, or reticuloendothelial system rather than persisting at the wound site [133,134].

The immune system is not solely destructive but also provides regenerative cues. MSCs can temper inflammation through the secretion of prostaglandin E2, IDO, and anti-inflammatory cytokines. EVs may carry microRNAs that reprogram macrophages toward a pro-regenerative M2 phenotype. The challenge is that these beneficial interactions occur in the same milieu that drives clearance [135,136].

A further concern is tumorigenesis. While MSCs themselves rarely undergo malignant transformation in vivo, their immunosuppressive and pro-angiogenic properties may create permissive niches for tumor progression. EVs can also transfer growth factors, microRNAs, or pro-angiogenic signals that, in dysregulated contexts, favor tumor-supportive microenvironments. Although robust clinical evidence of wound-associated oncogenesis from MSC/EV therapy is lacking, the theoretical risk necessitates cautious monitoring and stringent manufacturing standards.

Strategies to navigate these traps include cell engineering to reduce immunogenic markers, camouflaging EVs with membranes or biomaterial coatings, and controlled release systems that shield regenerative products during the most hostile early hours post-delivery [137,138]. In parallel, careful patient selection, oncological screening, and dose/time restrictions reduce the theoretical risk of tumorigenesis [139,140]. These approaches acknowledge that immune interaction is not an obstacle to be eliminated but a partner to be carefully choreographed.

#### Neuroimmune Crosstalk: A Hidden Determinant of Clearance and Scarring

The immune response to cell therapies is not solely dictated by local inflammatory cues, but also by neuroimmune circuits that provide higher-order regulation. Neuropeptides such as calcitonin gene-related peptide (CGRP) and Substance P modulate macrophage polarization, mast cell activation, and vascular permeability, thereby shaping whether transplanted products are cleared or integrated [141,142,143]. Similarly, vagal pathways exert anti-inflammatory tone via the cholinergic anti-inflammatory reflex, dampening excessive clearance and promoting tissue repair [144,145]. When these circuits are balanced, immune cells receive signals that support repair and tolerance. When these pathways are dysregulated, as in stress, chronic pain, neuropathy, circadian disruption, or systemic inflammation, the immune system becomes “mis-educated”: macrophages and mast cells adopt pro-inflammatory, hyper-clearance behaviors that strip MSCs and EVs before their regenerative cues can take effect [146].

Tricks to leverage neuroimmune control include pairing regenerative delivery with neuromodulatory adjuncts, such as vagus nerve stimulation or peptide-based neuromodulatory interventions, as well as temporal alignment of therapy with circadian windows when immune tone is more permissive. This underscores that clearance is not a purely immunological trap, but a failure of neuroimmune instruction, i.e., immune mis-education via neuroimmune crosstalk, and therefore a target for novel combinatorial strategies.

In the context of wounds, these maladaptive signals have tangible consequences. Substance P and CGRP released from damaged sensory nerves amplify vasodilation and neutrophil recruitment, perpetuating inflammation and accelerating clearance of transplanted products [143]. Conversely, impaired vagal tone—as seen in chronic stress, diabetes, or neuropathic states—removes an important anti-inflammatory brake, tipping the balance toward fibrosis and non-healing ulcers [145,147]. Thus, neuroimmune mis-education directly shapes the wound niche, determining whether it evolves into a permissive or hostile environment for regenerative therapies (Supplementary Table S5).

This principle extends beyond cutaneous wounds: in central nervous system injuries, maladaptive neuroimmune crosstalk contributes to micro-scarring that walls off axonal regrowth and prevents full spinal cord and nerve regeneration. The same mis-education that accelerates clearance in skin wounds also underlies the persistence of glial scars in the CNS, underscoring the universality of neuroimmune traps across tissues.

### 2.6. Safety Concerns: Regulatory, Economic, and Ethical Traps

No discussion of cell therapies can be complete without addressing the safety, regulatory, ethical, and economic frameworks that govern their translation. Biological traps undermine efficacy at the wound site; however, systemic traps—embedded in oversight, manufacturing, and patient access—often dictate whether promising interventions ever reach the clinic (Supplementary Tables S6 and S23).

Regulatory ambiguity remains a persistent barrier. Agencies such as the U.S. Food and Drug Administration (FDA) and the European Medicines Agency (EMA) broadly categorize MSCs and EVs as advanced therapy medicinal products (ATMPs) or biologics [148]. Precise definitions vary by jurisdiction, and borderline products such as stromal vascular fraction or conditioned medium blur the distinction between minimally manipulated tissue and engineered biologics. Potency assays for MSCs and EVs remain insufficiently standardized, leaving regulators without reliable benchmarks to assess efficacy or long-term safety.

Manufacturing and scalability amplify these challenges. GMP-compliant MSC expansion requires carefully controlled environments, validated release criteria, and qualified donor material, each layer adding cost and complexity. EV manufacturing is even less mature: low yields, batch-to-batch variability, and unresolved issues in cargo characterization undermine reproducibility [149,150]. Storage and distribution compound the problem, as MSCs and EVs typically require cold-chain logistics, which drives up costs and limits global accessibility.

Economic considerations intersect with these barriers. High production costs translate into therapies priced far beyond the reach of most health systems, especially for chronic wounds, which impose a disproportionate burden on older and socioeconomically disadvantaged patients. Without scalable and cost-effective production platforms, regenerative wound care risks remaining an elite intervention rather than a standard of care.

Ethical traps have further complicated the landscape, exploiting patient vulnerability, eroding public trust, and obscuring the progress of legitimate translational research. The rapid expansion of unregulated “stem cell clinics” offering poorly characterized products has led to safety incidents and high-profile warnings from the FDA, EMA, and scientific societies, emphasizing that no exosome product is currently authorized for wound healing or cosmetic use, and that unverified preparations pose risks of contamination, transmission of pathogens, and mislabeling of cellular or vesicular content. Similarly, the International Society for Cell and Gene Therapy (ISCT) and the International Society for Stem Cell Research (ISSCR) explicitly warn against ‘clinic-ready’ EV formulations lacking GMP manufacturing, potency testing, and sterility validation. These guidance documents highlight a critical trap: patients often receive products with unknown provenance, variable dosing, or adulterants that may include microbial EVs, endotoxins, or non-sterile excipients. For the same reason, off-label or do-it-yourself use of DMSO, herbal mixtures, or online-purchased EV products should be strongly discouraged. Although DMSO is discussed in this review within the context of emerging mechanistic frontiers, it is not clinically validated for topical or regenerative wound applications, and its membrane-penetrating properties raise the risk of delivering unintended toxins or pathogens when used outside controlled laboratory conditions. Similarly, unstandardized herbal extracts carry dose-dependent cytotoxicity, solvent penetration risks, and unpredictable drug interactions, and their use outside clinical oversight may amplify rather than mitigate wound pathology. Ensuring that regenerative therapies originate from regulated, GMP-compliant manufacturing, and avoiding unauthorized or improvised interventions, is therefore essential for safeguarding patient safety and scientific integrity.

Several tricks are emerging to address these systemic barriers, emphasizing safety-by-design and harmonization. Proactive dialog with regulators enables early alignment with evolving standards, while initiatives such as MISEV (Minimal Information for Studies of Extracellular Vesicles) promote transparency and reproducibility. Bioreactor-based closed systems enhance scalability and reduce the risk of contamination. Cost-mitigation strategies, such as allogeneic “off-the-shelf” cell sources, immortalized MSC lines, and lyophilized EV formulations stable at ambient temperature, offer realistic pathways to broader accessibility. Equally critical are transparency and patient engagement: registries, long-term follow-up protocols, and clear communication help rebuild trust and distinguish rigorous science from commercial opportunism. Patient education may counteract misinformation and strengthen ethical practice.

In this sense, safety, regulation, and economics are not peripheral issues but central determinants of translational success. Only by addressing these systemic traps can stem cell and exosome therapies in wound healing evolve from experimental promise to globally impactful clinical reality.

## 3. Case Studies: Clinical Manifestations of Traps and Tricks

The preceding sections outlined how stem cell and exosome therapies encounter universal traps—microenvironmental hostility, infection control dilemmas, poor retention, scarring bias, immune clearance, and systemic barriers. These concepts become most tangible when viewed through clinical case studies, where the interplay of traps and tricks unfolds in practice.

Case studies illustrate that no single therapy fails or succeeds in isolation: outcomes are determined by how regenerative payloads interact with the specific niche context. Burns exemplify acute environments dominated by necrosis, inflammation, and the scarring paradox. Chronic wounds illustrate the opposite extreme: a pathological equilibrium sustained by biofilms, immune mis-education, and metabolic dysfunction. Grafting strategies show how MSCs and EVs can augment or undermine one of the oldest surgical approaches, while oral ulcers highlight the unique challenges of a microbiome-rich, mechanically dynamic niche.

These case studies underscore that clinical translation does not merely depend on adding stem cells or exosomes, but on recognizing and redesigning the niches they enter. Each scenario reveals traps that have historically derailed regenerative strategies and tricks that are beginning to transform them, setting the stage for the more unconventional and emerging approaches discussed in the next section.

To contextualize these patterns within the broader clinical literature, a scoping summary of representative MSC and MSC-derived EV clinical trials across diabetic foot ulcers, venous leg ulcers, burns, and pressure ulcers is provided in Supplementary Tables S7–S10 and S24. To ensure measurable outcomes, scar progression should typically be evaluated using validated clinical instruments, such as the Patient and Observer Scar Assessment Scale (POSAS) and the Vancouver Scar Scale (VSS), alongside elasticity metrics (e.g., cutometer-derived stiffness). POSAS and VSS scores should typically be collected at 8–12 weeks and again at 3–6 months to allow for a clear distinction between normotrophic scars and hypertrophic or keloid pathways in clinical trials and translational studies.

### 3.1. Burns

Burns represent one of the most challenging contexts for regenerative therapy. Extensive tissue necrosis, vascular thrombosis, and high inflammatory burden create a profoundly hostile niche for transplanted MSCs or EVs. The primary traps include overwhelming oxidative stress, disrupted vascular supply, and the paradoxical need for aggressive infection control. Silver-based dressings, while indispensable for microbial suppression, exert cytotoxic effects on regenerative products, compromising MSC survival and destabilizing EV cargo. Clinically, this manifests as inconsistent outcomes, with studies reporting accelerated epithelialization, limited benefit, or the development of rigid, hypertrophic scars [151,152].

The scarring bias is particularly pronounced in burns. Even when stem cell or exosome therapy accelerates closure, the repair process often defaults to fibrosis, producing hypertrophic or keloid scars that restrict mobility and impair quality of life. This duality exemplifies the Scarring Paradox: therapies can speed healing yet inadvertently reinforce fibrotic trajectories if the niche remains dysregulated.

Tricks emerging in this space focus on protective delivery and anti-fibrotic modulation. Encapsulation of MSCs in hydrogels or collagen scaffolds enhances retention and shields against oxidative insult. Sequential dressings—initial silver for microbial control, followed by biocompatible carriers—balance infection management with regenerative support [153,154,155]. Anti-fibrotic adjuvants (e.g., losartan, quercetin, verteporfin) can be paired with MSCs/EVs to dampen scar-promoting pathways, while biomaterial cues (aligned nanofibers, soft scaffolds) guide organized tissue regeneration. The approaches illustrate how burn therapy moves from simply “adding stem cells” to engineering the wound environment for regenerative success (Supplementary Table S7).

### 3.2. Chronic Wounds

Chronic wounds, including diabetic foot ulcers, venous leg ulcers, and pressure sores, represent one of the most compelling but also one of the most frustrating targets for stem cell and exosome therapies. Unlike acute injuries, chronic wounds are sustained by a pathological equilibrium: persistent low-grade infection, ischemia, and metabolic dysfunction lock the tissue into an inflammatory state that resists progression through typical phases of healing [156,157]. For MSCs and EVs, this environment represents a particularly intractable trap.

The microenvironment is dominated by biofilm persistence. Even when microbial burden is reduced, biofilms maintain proteolytic activity, oxidative stress, and pH instability, degrading exosomal cargo and impairing MSC function [158,159]. Immune mis-education is amplified in chronic wounds, where macrophages and neutrophils remain locked in pro-inflammatory states. Neuroimmune contributions, such as impaired vagal tone in diabetes or neuropathy, further tilt the balance toward clearance rather than integration [146,156].

Delivery and dosing challenges are magnified in this setting. Topical MSC suspensions or exosome washes are quickly lost to exudate, while injected products succumb to hypoxia and nutrient deprivation [160,161]. The Dosing Paradox is especially pronounced: escalating doses in hopes of overcoming the hostile niche only increase washout, metabolic stress, and immune clearance. Instead of saturating the system, regenerative therapies risk being drowned out by pathological feedback loops [162,163].

Simultaneously, chronic wounds expose the Scarring Paradox in reverse. While fibrosis impairs function in acute wounds and burns, chronic wounds often fail to mount adequate fibrotic closure, leading to perpetual open lesions. Here, the trap is not excessive scarring but the inability to trigger regenerative fibrosis at all, underscoring how context determines whether MSCs and EVs suppress or enable repair.

Tricks emerging in this space aim to reprogram the wound rather than simply add regenerative payloads. Strategies include disrupting biofilms with quorum-sensing inhibitors or bacteriophages, restoring vascular supply through pro-angiogenic scaffolds, and pairing MSC/EV therapy with metabolic modulation (e.g., glucose control and oxygen therapy). Fractionated dosing, combined with protective carriers such as hydrogels or microneedle patches, enhances retention while reducing paradoxical clearance. Finally, adjunctive approaches represent unconventional yet promising tricks to tilt the chronic wound niche back toward regeneration (Supplementary Table S8).

### 3.3. Grafting Strategies

Skin grafting remains a cornerstone in the treatment of large or deep wounds, yet graft success is often compromised by ischemia, infection, and integration mismatch. For stem cell and exosome therapies, grafting presents both an opportunity and a trap: the graft surface provides a defined substrate for cell or vesicle application, but the same hostile conditions that threaten graft survival also undermine regenerative payloads [164].

Engraftment failure is a recurrent challenge. Ischemic zones beneath the graft limit oxygenation and nutrient diffusion, leading to early necrosis of both graft tissue and transplanted MSCs [165,166,167]. Inflammatory cues amplify this process, with neutrophil-driven proteolysis degrading exosomal proteins and ECM components. Even when the graft survives, integration mismatch—a disorganized alignment of collagen and dermal-epidermal junctions—yields functionally compromised tissue that is prone to contraction and fibrosis [168].

Here, the Scarring Paradox is again evident: while MSCs and EVs can improve graft take and accelerate epithelialization, in dysregulated environments, they may accelerate fibrotic remodeling, producing stiff, contractile scars that reduce functional outcomes.

Tricks to improve graft success center on creating supportive interfaces. Pre-seeding scaffolds or graft matrices with MSCs/EVs before application increases retention and shields against early ischemic stress. Pro-angiogenic scaffolds (VEGF-laden hydrogels, oxygen-releasing biomaterials) enhance perfusion and reduce necrosis [169,170]. Exosomes engineered with targeting ligands improve homing within graft tissue, while mechanical conditioning of graft matrices (aligned nanofibers, elastic supports) promotes organized tissue integration [171]. Clinically, sequential strategies such as applying MSC/EV carriers post-graft stabilization rather than at the ischemic outset are beginning to show promise in balancing early survival with long-term regenerative outcomes (Supplementary Table S9).

### 3.4. Oral Ulcers

Oral ulcers, ranging from recurrent aphthous ulcers and traumatic lesions to radiation- or chemotherapy-induced mucositis, represent a distinct clinical niche for regenerative therapies. Unlike cutaneous wounds, the oral cavity presents a moist, microbiome-rich, and mechanically dynamic environment that poses unique traps on stem cell and exosome delivery. The oral mucosa is exposed to constant mechanical shear, rapid fluid washout, and the highest microbial density outside the gut. As a result, transplanted MSCs or EVs are quickly diluted, degraded, or competitively suppressed. Clinical attempts with topical suspensions or gels often show only transient benefit, with poor persistence of regenerative cues [172,173].

Beyond these physical challenges, oral ulcers illustrate the Microbiota-Exosome Axis in real time. Dysbiotic bacterial and fungal EVs in the oral cavity compete directly with therapeutic vesicles, skewing macrophage responses and prolonging inflammatory tone [174,175]. Latent viral infections, such as herpesviruses embedded in oral mucosa, can further distort EV cargo, creating a vesicular “signal interference” pattern that undermines MSC/EV function [176,177,178]. This phenomenon echoes the broader principle outlined in Section 2.4: microbiota are not a passive background, but active modulators of wound outcomes through vesicle-mediated communication [179].

Tricks to counter these traps include mucoadhesive carriers, which anchor regenerative vesicles at the lesion site; probiotic lozenges or rinses, which can rebalance microbial EV output; and experimental strategies such as engineered MSC-derived EVs designed to resist microbial uptake [180]. Oral ulcers, therefore, serve as a didactic example of how regenerative therapies fail when the microbiota-exosome dialog is unbalanced, and how reframing treatment around this axis may open new therapeutic avenues.

From a biological perspective, oral ulcers illustrate the Dosing Paradox and aspects of Neuroimmune Mis-education. Escalating doses of MSCs or EVs in liquid suspensions rarely overcome clearance, instead amplifying washout [181,182]. Meanwhile, pain-associated neuropeptides, such as Substance P, heighten local inflammation, thereby worsening immune clearance of transplanted cells and vesicles.

Tricks in this domain focus on stabilizing regenerative products in the oral niche. Mucoadhesive carriers, including chitosan, hyaluronic acid, and alginate hydrogels, prolong contact time and protect vesicles from enzymatic degradation. Novel delivery systems, such as dissolvable oral patches, lozenges, or thin films, offer controlled release and patient-friendly formats [183]. Adjuncts such as antimicrobial peptides or probiotic formulations can mitigate dysbiosis, while pH-buffering biomaterials stabilize vesicle integrity. Experimental approaches include combining MSC/EV carriers with pain-modulating agents to reduce neurogenic inflammation and improve regenerative integration.

By reframing oral ulcers as a dynamic but engineerable niche, these strategies illustrate how classical traps, such as washout, microbiome interference, and neuroimmune amplification, can be reconfigured into opportunities for innovation in localized regenerative therapy (Supplementary Table S10).

### 3.5. The Predictive Role of Triads

Clinical heterogeneity in wound healing outcomes suggests that therapeutic success or failure cannot be explained by local context alone. These case studies illustrate how the same therapeutic payload—MSC transplantation or exosome delivery—can succeed in one wound type but falter in another. Burns overwhelm transplanted cells through acute ROS surges, chronic ulcers neutralize therapies through microbial competition, and keloids subvert regenerative vesicles into pro-fibrotic signals. Such heterogeneity is often read as inconsistency of the treatment, but a deeper view suggests that each wound type is defined by a characteristic constellation of traps—a pattern that is later framed as a triad of systemic regulators. This observation sets the stage for Section 6, where these patterns are formalized into a predictive matrix that links wound type, triad dysfunction, and vesicle signature. By transitioning from anecdotal variability to systematic mapping, what appears as inconsistency begins to resolve into a hidden order.

### 3.6. Pattern Recognition: From Clinical Lessons to Strategic Directions

The case studies examined above reveal a critical pattern: the failure of stem cell and exosome therapies is not random but reflects predictable interactions between regenerative payloads and specific niche contexts. Burns overwhelm therapies through acute oxidative stress (Section 3.1), chronic wounds neutralize them through microbial competition (Section 3.2), grafting failures expose ischemic vulnerabilities (Section 3.3), oral ulcers demonstrate rapid washout dynamics (Section 3.4), and DMSO illustrates how ancillary agents can either amplify or undermine therapeutic goals (Section 5.7). These diverse manifestations of failure share common mechanistic threads—hostile microenvironments, poor retention, immune clearance, and the paradoxical conversion of regenerative signals into pathological ones. Importantly, each case study also points toward strategic interventions that could transform these traps into therapeutic opportunities. The recognition that traps are not insurmountable barriers but addressable system vulnerabilities has catalyzed a new generation of innovations. These emerging tricks, examined in the following section, demonstrate how the lessons of failure can be systematically converted into strategies for success.

## 4. Emerging Tricks

The traps and paradoxes identified in Section 2, illustrated by case studies in Section 3, reveal why stem cell and exosome therapies have struggled to achieve consistent clinical success. They also point toward a critical insight: many of these obstacles can be addressed with incremental but pragmatic innovations that refine how regenerative payloads are prepared, delivered, and matched to patients. Unlike the more visionary strategies considered later in Section 5, these emerging tricks are already being translated into preclinical pipelines and early clinical trials.

These strategies fall into several domains. Preconditioning and biomaterial carriers (Section 4.1) enhance MSC and EV survival by buffering against oxidative stress, hypoxia, and immune clearance. Exosome engineering and filtration (Section 4.2) address the problem of double agency by purifying therapeutic vesicles and reprogramming their cargo. Anti-fibrotic and senolytic combinations (Section 4.3) counter the scarring paradox, rebalancing regenerative signaling in favor of functional tissue restoration. Finally, patient stratification and personalization (Section 4.4) address the issue of failed cell therapies, acknowledging that heterogeneous wounds necessitate individualized interventions.

These emerging tricks demonstrate that translation does not need to await speculative technologies: many of the critical traps can already be addressed via systematic, context-aware strategies that fundamentally reconceptualize therapeutic preparation, delivery, and stratification. Section 5 will then expand to consider more unconventional horizons, where traps are reframed into programmable variables that open entirely new directions for regenerative medicine.

### 4.1. Preconditioning and Biomaterial Carriers

One of the most persistent traps in regenerative wound therapy is the fragility of transplanted MSCs and EVs in hostile niches. Once delivered into the wound bed, cells are rapidly lost to oxidative stress, hypoxia, and immune clearance, while vesicles diffuse freely, are phagocytosed, or absorbed into dressings [3,184]. Even when high doses are administered, retention remains poor, often exacerbating washout and amplifying clearance. This dilemma underscores the need for strategies that not only increase the initial payload but also prolong its survival and functional persistence.

A promising line of work has reframed stress itself as a tool. The paradox, framed as the Preconditioning Paradox, reflects that conditions typically destructive in vivo, such as hypoxia, oxidative bursts, or cytokine exposure, can, when carefully applied in vitro, enhance resilience. Hypoxic preconditioning, for example, shifts MSC metabolism toward glycolytic flexibility and enriches their secretome with angiogenic and anti-inflammatory factors [89,185,186]. Similarly, oxidative or cytokine priming can bias EV cargo toward microRNAs and proteins that dampen fibrosis and promote repair. In this sense, the very stressors that undermine cell therapy in the wound can be redeployed as tricks to prepare cells and vesicles for survival [170].

Biomaterial science has advanced to provide protective carriers that recreate aspects of a stabilizing niche [185]. Hydrogels and ECM scaffolds prolong MSC and EV retention while permitting the gradual release of signals. Conductive and ion-responsive materials buffer polarity and redox fluctuations, counteracting electrical and oxidative noise. Newer delivery platforms such as sprayable films, microneedle patches, and 3D-printed scaffolds add spatial and temporal precision, ensuring that regenerative cues are deposited where and when they are most needed [187,188,189].

The field is shifting from brute-force dosing toward smarter preparation and protection. By preconditioning MSCs and EVs to withstand stress and embedding them in engineered carriers that stabilize the wound niche, therapies are no longer passively exposed to hostile environments but actively insulated and guided, turning destructive forces into resilience-building cues (Supplementary Table S11).

### 4.2. Exosome Engineering and Filtration

Exosomes have been celebrated as safer, cell-free surrogates of MSC therapy; however, the trap of double agency shadows their translation. In regenerative niches, exosomes carry microRNAs and proteins that promote angiogenesis, dampen inflammation, and modulate fibroblast activity [190]. In dysregulated environments, however, they may propagate pathological cues—driving senescence, fibrosis, or even tumor support [191]. The recognition that vesicles can act as therapeutic agents and pathological messengers reframes the challenge: progress does not depend on assuming exosomes are intrinsically beneficial, but on learning to separate and redesign their signals.

The Exosome Paradox arises here: the exact mechanism that gives EVs their versatility—the ability to mirror the physiological state of their parent cells—also makes them vulnerable to transmitting harmful instructions. MSCs expanded under stress release senescence-associated EVs rich in pro-inflammatory cargo; microbiota shed vesicles that mimic host signals and compete with regenerative cues; fibroblast-derived vesicles can reinforce collagen cross-linking [192]. Left unfiltered, these vexosomes obscure the therapeutic signal.

Tricks to counter this double agency are emerging on two fronts. The first is filtration and selection [193]. Techniques such as size exclusion chromatography, immunoaffinity capture, and microfluidic sorting can enrich regenerative vesicles while excluding microbial or senescence-associated subtypes [194]. Novel filtration devices now allow selective removal of vesicles with fibrotic or pro-inflammatory signatures, reframing EV therapy as an engineered product rather than a crude extract. The second frontier is engineering. MSCs can be preconditioned or genetically modified to package specific therapeutic microRNAs, surface ligands, or proteins into their vesicles [195,196,197]. EVs themselves can be directly loaded with cargo or coated with targeting ligands to improve homing and reduce off-target clearance.

These advances suggest that exosome therapy is shifting from descriptive biology to programmable biotechnology. By acknowledging the double agency trap and leveraging filtration and engineering tricks, the field is moving toward a future where vesicles are carriers of context and instruments of design, capable of delivering precision regenerative instructions. Compared with whole-cell transplantation, exosome-based therapy offers a more standardized and ethically streamlined pathway to clinical use, converting the regenerative instructions of MSCs into a defined, cell-free biopharmaceutical format (Supplementary Table S12).

### 4.3. Anti-Fibriotic and Senolytic Combinations

One of the most tenacious traps in wound healing is the tendency of MSCs and EVs to be diverted from regeneration into fibrotic signaling. In supportive niches, they suppress scarring. In hostile niches, they may paradoxically reinforce collagen deposition and contracture. This Scarring Paradox reflects the duality of wound repair: the same fibroblast activation that closes a defect also risks sealing it with non-functional tissue [198,199].

Therapies aimed at breaking this paradox increasingly turn toward anti-fibrotic and senolytic combinations. Fibrosis and cellular senescence are intimately linked: senescent fibroblasts and immune cells secrete a pro-fibrotic secretome, rich in TGF-β, IL-6, ECM cross-linkers, that biases healing toward scar formation [200]. MSC-derived EVs may dampen this cascade in some contexts, but in others, they are reprogrammed by the fibrotic milieu, amplifying instead of reversing it. The trap, therefore, lies in the biology of scars and in the dynamic feedback loop between therapeutic vesicles and senescent cells [197,201].

The emerging trick is to pair regenerative therapies with anti-fibrotic or senolytic agents. Small molecules such as pirfenidone or nintedanib, as well as natural compounds like quercetin, have been shown to reduce fibroblast activation or selectively clear senescent cell populations. When combined with MSCs or EVs, they rebalance the wound environment, allowing regenerative cues to dominate. Experimental approaches include co-delivery of senolytic-loaded nanoparticles with EVs, embedding anti-fibrotics into hydrogel scaffolds, or engineering vesicles to carry anti-fibrotic microRNAs. The guiding principle is synergy: regeneration is more effective when fibrosis and senescence are actively suppressed rather than left to counteract therapy.

These strategies underscore that wound healing is not a binary between scar and regeneration but a spectrum shaped by cellular cross-talk. By targeting the senescence-fibrosis loop, anti-fibrotic and senolytic combinations reveal how traps can be neutralized not by more cells, but by smarter adjunctive programming of the niche (Supplementary Table S13).

### 4.4. Patient Stratification and Personalization

A recurring trap in the clinical translation of MSC and EV therapies is the failure of randomized controlled trials (RCTs) to replicate the striking outcomes seen in preclinical models or small, uncontrolled studies [202]. This gap is often interpreted as evidence that the therapies themselves are weak or inconsistent [203]. However, the deeper trap lies in patient heterogeneity. Chronic wounds are not a single entity but a spectrum of pathologies: diabetic ulcers with metabolic stress, venous ulcers with vascular insufficiency, pressure ulcers with ischemia and biofilm dominance. Delivering the same regenerative therapy across these divergent contexts dilutes potential benefit, ensuring that “average” trial outcomes conceal meaningful responses in subgroups.

This challenge is captured in what might be called the Stratification Paradox: the more heterogeneous the patient cohort, the less likely a uniform therapy will be successful. However, the more precisely therapies are matched to the context, the greater their effect becomes. The problem is not necessarily the therapy itself, but rather the misalignment between the treatment and the patient’s profile.

Emerging tricks focus on patient stratification and personalization. Biomarker-guided selection is one avenue, using circulating cytokines, EV signatures, or wound fluid metabolites to identify patients likely to benefit from MSC/EV therapies [204]. Another approach is to classify wounds by their dominant trap profiles, such as oxidative stress, infection, fibrosis, or immune mis-education, and tailor adjuncts accordingly [205,206]. Digital tools, including machine learning applied to wound images and multi-omic datasets, provide scalable methods for matching regenerative payloads with specific wound phenotypes. Personalized dosing regimens, such as fractionated applications, timing with circadian windows, or niche-aware delivery, extend this precision.

The take-home lesson is that regenerative therapy cannot be a one-size-fits-all approach. Stratification does not reframe failed RCTs as evidence against MSCs or EVs, but as demonstrations that heterogeneity is the real obstacle. By recognizing patient-specific trap constellations and tailoring therapy accordingly, regenerative medicine can shift from blunt interventions to precision strategies (Supplementary Table S14).

## 5. Emerging Frontiers—Horizon-Level Concepts

The traps and paradoxes described in earlier sections reveal why conventional applications of MSCs and EVs often falter. However, they also point toward a more profound insight: wound biology is not only reactive, but inherently programmable. Once hostile microenvironments, bioelectric collapse, or vexosomes are recognized as systemic traps, they can be reframed into targets for deliberate modulation. This recognition has spurred a wave of emerging and often unconventional strategies that move beyond incremental optimization into truly paradigm-shifting tricks.

These strategies share three features. First, they extend beyond the cell itself, focusing on the niche signals: electrical, circadian, microbial, hematologic, or ecological that determine whether regenerative instructions are received, amplified, or silenced. Second, they embrace unconventional adjuncts, from herbal bioactives and small molecules to neuromodulatory interventions, digital twins, and AI-guided wound mapping. Third, they aim for programmability, treating the wound not as a static site of damage but as a dynamic, information-rich system whose polarity, timing, immune tone, and vesicle traffic can be tuned.

In this section, several emerging tricks are highlighted. Bioelectric reset demonstrates how electrical disorganization can be countered with precision stimulation and conductive scaffolds. Herbal paradoxes illustrate how natural compounds, long dismissed as anecdotal, may function as programmable regulators of vesicle cargo, niche stability, and microbiota interactions. Herbal modulators of the stem cell niche bridge traditional and modern approaches, may offer affordable adjuncts that stabilize wound environments and potentiate MSC/EV therapies. Circadian synchronization aligns therapy delivery with permissive immune and repair windows. Digital twins and systems modeling exemplify how computational frameworks can integrate traps and paradoxes into predictive guides for therapy. Speculative adjuncts such as erythrocyte-derived ACA plasticity or cross-kingdom vesicle modulation suggest that even radical hypotheses can yield insights when reframed within the traps-paradoxes-tricks continuum. Finally, the example of DMSO demonstrates how even ancillary compounds, long relegated to cryostorage, can act as hidden determinants of therapeutic success or failure.

Note on scope: The concepts explored in Section 5.1, Section 5.2, Section 5.3, Section 5.4, Section 5.5, Section 5.6 and Section 5.7 represent emerging or horizon-level exploratory frontiers that extend beyond currently validated practice. They are presented to stimulate hypothesis-driven research, not to provide clinical recommendations. The evidentiary status, supporting rationale, and cautionary notes for each theme are summarized in Supplementary Horizon Tables S25–S28.

### 5.1. Bioelectric Reset: Reprogramming the Electrical Niche

The loss of endogenous electric fields after injury is one of the most subtle but consequential traps in wound biology [207]. Endogenous transepithelial fields of 40–200 mV/mm usually guide keratinocytes, fibroblasts, and endothelial cells via electrotaxis [208]. During normal healing, epithelial ion flow generates an endogenous wound current of approximately 10–100 µA/cm^2^, which gradually declines as re-epithelialization restores tissue continuity. This current corresponds to the measured lateral electric fields of ~40–200 mV/mm because voltage gradients and current densities are linked by the electrical conductivity of wounded tissue. When epithelial integrity collapses, polarity is disrupted and the wound becomes electrically disorganized, generating incoherent migration signals that delay closure. This impairs intrinsic healing and undermines the integration and delivery of transplanted MSCs and EVs, which rely on spatial cues to function effectively (Supplementary Tables S15, S27 and S28).

The trick lies in restoring a coherent electrical milieu, a concept referred to as bioelectric reset [209]. Controlled external stimulation can re-establish polarity and guide cellular migration, effectively “rebooting” the wound niche. Animal and early clinical studies suggest that microampere-level currents (10–100 μA/cm^2^) or applied fields in the range of 40–200 mV/mm can accelerate re-epithelialization, enhance angiogenesis, and reduce infection [210,211]. Conductive biomaterials—such as graphene scaffolds, polypyrrole films, or ion-conducting hydrogels—extend this principle by embedding polarity into the dressing, providing continuous directional cues.

MSC- and EV-based therapies are particularly well-suited to synergize with bioelectric reset [212]. Electrical stimulation enhances MSC survival, promotes exosome release, and biases EV cargo toward pro-regenerative microRNAs. Conversely, EVs can stabilize membrane channels and improve the consistency of cellular electrotaxis under stimulation, creating a feedback loop between bioelectric cues and regenerative payloads. This reciprocity reframes bioelectric reset not as an isolated adjunct but as an amplifier of cell-based strategies.

The main challenge is precision. Overstimulation risks cytotoxicity, while inadequate or misaligned fields fail to impose order [213,214]. Future strategies include closed-loop bioelectronics systems that measure local wound impedance or voltage gradients and dynamically adjust stimulation to maintain physiological ranges. Such programmable systems could transform electrical disorganization from a hidden liability into a controllable variable, aligning with the broader theme of traps becoming programmable levers for paradigm shifts. Emerging hybrids combine bioelectric reset with optogenetic tools, using light-gated ion channels to impose spatiotemporal precision. This bioelectric-optogenetic horizon suggests that electrical reprogramming could eventually be orchestrated with single-cell and millisecond resolution.

### 5.2. Herbal Paradox: From Anecdote to Programmable Regulators of Stem Cell Niche

Although valued empirically, the deployment of herbal compounds as modulators of the stem-cell niche is a frequently overlooked practical strategy to stabilize regenerative therapies [215,216]. Unlike purely speculative frameworks, this approach is grounded in a growing body of preclinical and clinical evidence showing that botanical extracts can mitigate oxidative stress, temper inflammation, and promote angiogenesis—precisely the traps that undermine MSC- and EV-based therapies [217]. The trap lies in perceiving herbal agents as anecdotal or unscientific: variability in preparation, inconsistent dosing, and limited mechanistic understanding have slowed their integration into mainstream regenerative protocols. However, when studied systematically, many herbal bioactives demonstrate clear relevance to wound biology. Curcumin suppresses NF-κB activation and reduces oxidative spikes that kill transplanted MSCs [218,219]; Centella asiatica extracts stimulate collagen remodeling and angiogenesis [220,221,222]; green-tea polyphenols reduce protease activity, protecting ECM integrity [223,224,225]; aloe vera and plantain extracts provide hydration and antimicrobial buffering, stabilizing the wound niche for regenerative cues.

The deeper Herbal Paradox is that natural agents, often dismissed as low-tech and inconsistent, can intervene at the same systemic choke points that confound advanced MSC/EV therapies. The very simplicity that relegates them to the periphery may conceal programmable properties capable of reshaping vesicle cargo, microbial balance, and regenerative timing. Polyphenols, flavonoids, and terpenes can reprogram EV cargo, biasing vesicles toward anti-inflammatory microRNAs or anti-fibrotic proteins [226,227]. Plant-derived metabolites interact with the microbiota, altering microbial-EV profiles and reducing pathobiota interference [228]. Some compounds act as vectors, crossing membranes and transporting other bioactive compounds deep into tissues [227,229]. In this sense, herbal compounds are not merely buffers against hostile environments, but rather active shapers of vesicle-mediated communication [230,231].

The trick is to harness this capacity deliberately. Herbal bioactives can be integrated into MSC culture systems to precondition secretomes, embedding desired signals into exosomes before delivery [232]; co-formulated with EVs to stabilize cargo; or delivered systemically to recalibrate microbial ecology. Coupling herbal compounds with programmable carriers, such as nanoparticles, microneedle patches, or hydrogels, allows for targeted deployment with reproducible dosing [225]. Reframing herbal medicine as an informational intervention, capable of modulating EV traffic, immune tone, and microbial competition, positions what once seemed peripheral as a central element of regenerative programming.

Importantly, herbal systems should not be reduced to single “active” compounds [233,234,235,236]. Their efficacy often emerges from synergistic networks, where one constituent buffers toxicity, another stabilizes delivery, and others trigger downstream cascades [237,238]. This systemic synergy is not a liability but a hidden architecture that can be deliberately harnessed for regenerative healing. Finally, herbal systems intersect with the broader vesicle ecology. Plant- and fungal-derived EVs can cross into mammalian biology, influencing immunity and niche tone, suggesting a frontier of cross-kingdom vesicle communication that may be co-opted for regenerative control (Supplementary Table S16).

### 5.3. Circadian Synchronization: Time as a Therapeutic Lever

Healing unfolds across space and time. The circadian clock orchestrates immune activity, redox balance, vascular tone, and cell proliferation, creating daily oscillations that shape how wounds respond to regenerative cues. However, most regenerative interventions are delivered without regard to this temporal architecture, a trap defined as temporal blindness.

The Circadian Paradox is that the very rhythms that undermine therapy when ignored can amplify it when harnessed. Neutrophil infiltration peaks during rest phases, fibroblast migration is enhanced during active phases, and vascular perfusion oscillates across day-night cycles. Administering MSCs or EVs during pro-inflammatory windows accelerates clearance, while delivering them during permissive phases can double their effective integration. Time is, therefore, not a neutral backdrop but a determinant of therapeutic fate.

Unconventional strategies are beginning to treat time as a programmable variable [239]. MSCs can be preconditioned in vitro with circadian cues—light/dark cycles, melatonin pulses, or glucocorticoid rhythms, producing EVs biased toward anti-inflammatory or pro-angiogenic cargo. In vivo, chronotherapy aligns delivery with patient-specific circadian windows, aided by wearables and digital chronobiology platforms that map immune and metabolic rhythms. Adjunctive interventions, such as melatonin supplementation, vagal nerve stimulation, or cortisol modulators, can help restore disrupted clocks in chronic wounds, stress, or diabetes, thereby creating an internal environment more receptive to regenerative signals.

The boldest implication is that circadian synchronization reframes regenerative therapy as a temporal dialog rather than a static intervention. Wounds may be programmable through cells, molecules, and niches, and through alignment with the body’s intrinsic oscillations. Time itself becomes a trick: a lever for precision healing that transforms the trap of temporal blindness into an opportunity for regenerative control (Supplementary Table S17).

### 5.4. Digital Twins and Predictive Simulation

Wound healing has traditionally been evaluated retrospectively: clinical scoring, histological snapshots, or delayed endpoints. This retrospective approach perpetuates traps by obscuring the dynamic interconnection of cells, vesicles, microbiota, and host physiology. The result is a therapy delivered into uncertainty—without clarity on which traps dominate in a given patient or how interventions shift healing trajectories.

The Digital Twin concept offers a radical departure. Instead of relying on averages or trial and error, a digital twin creates a patient-specific, computational replica of the wound system. By integrating clinical data, imaging, molecular biomarkers, and even wearable-derived metrics of perfusion or circadian rhythm, these models simulate how a wound evolves under different interventions [240]. In effect, digital twins expose traps in real-time and allow for predictive testing of tricks before they are deployed.

The paradox here lies in the Predictive Simulation Paradox: the very complexity that makes wounds unpredictable can, when adequately modeled, become a source of precision. Far from being noise, the multiple layers of data—immune tone, microbial load, EV cargo, or electrical signals—can be integrated into dynamic systems models that reveal when and how regenerative therapies succeed or fail.

Tricks to harness this vision are emerging. Machine learning models can already classify chronic versus acute wounds with accuracy exceeding that of human specialists [241]. Multi-scale simulations have begun to capture bioelectric gradients and predict the dynamics of re-epithelialization [242]. The integration of microbiome sequencing with EV profiling suggests the development of stratification matrices that can be embedded into predictive dashboards. In the near future, AI-guided twins may recommend optimal timing for MSC/EV administration, identify when to combine with senolytics or antimicrobials, and forecast whether regenerative cues are likely to persist or collapse [243,244].

Such frameworks recast wound healing not as a static outcome but as a programmable system. Digital twins transform the traps of unpredictability and heterogeneity into opportunities for simulation-driven personalization, turning uncertainty into a design space for regenerative care. Beyond simulation, AI is now entering compound discovery. Machine learning platforms are identifying novel molecules and combinations predicted to modulate EV cargo, fibroblast activation, or metabolic resilience—extending digital twins from predictive tools into engines of therapeutic design, ushering in silico “virtual pharmacology” (Supplementary Table S18).

### 5.5. Erythrocyte Plasticity as an Unconventional Horizon

Among intriguing concepts in regenerative medicine is the proposal that erythrocytes, long considered terminally differentiated, may harbor latent plasticity [245]. Evidence suggests that erythrocyte glycosylphosphatidylinositol (GPI)-anchored glycoproteins such as ACA can, under certain conditions, influence progenitor cell behavior and induce pluripotency-associated markers [246,247,248]. While this does not demonstrate that mature enucleated erythrocytes themselves regain proliferative capacity, it raises the possibility that erythrocyte-associated proteins participate in regenerative signaling. If validated, this would redefine erythrocytes not only as oxygen carriers but as a vast, circulating reservoir of latent regenerative potential.

The trap is the assumption of terminality—that mature red blood cells, lacking nuclei, are inert passengers in systemic physiology. This dogma has diverted attention away from subtler capacities such as membrane signaling, vesicle release, and possible reprogramming under stress [249]. By ignoring these possibilities, regenerative biology may be overlooking one of the most abundant and accessible cellular platforms in the body.

The paradox here is that cells designed for maximal specialization (oxygen transport) may conceal maximal plasticity. The very feature that defines erythrocytes—the absence of nuclei—has been interpreted as a barrier, yet it could enable them to serve as pliable vehicles for regenerative programming through surface-anchored proteins, vesicle exchange, and context-driven plasticity.

Tricks to explore this unconventional horizon remain speculative but conceptually powerful. Erythrocytes could be leveraged as bioreactors for regenerative vesicles, carrying therapeutic signals systemically and delivering them to wound sites. Their membrane properties might be engineered to bias EV cargo or interact with immune checkpoints [250]. Although highly speculative, ACA-driven mechanisms suggest a precedent for erythrocyte-associated factors influencing regenerative outcomes. Though experimental validation is scarce, the hypothesis reframes red cells from inert scaffolds of oxygen transport into potential participants in regenerative orchestration.

If proven, erythrocyte plasticity would expand the stem cell paradigm beyond specialized niches, positioning the circulatory system as a latent regenerative organ. Even as a speculative construct, it illustrates how unconventional thinking can transform assumptions into opportunities—demonstrating that the line between trap and trick often lies in perspective (Supplementary Table S19).

### 5.6. Mechanobiology and Metabolic Horizons

Wounds are not only biochemical and electrical systems but also profoundly mechanical and metabolic environments. Chronic wounds exhibit abnormal stiffness, shear, and tension, which bias fibroblasts toward the formation of scars. Their cellular metabolism is trapped in glycolytic bias and mitochondrial dysfunction, limiting the energy supply and flexibility needed for regeneration. These overlooked domains represent emerging horizons where unconventional tricks may reshape therapeutic outcomes.

**Mechanobiology.** The trap lies in uncontrolled mechanical stress. Fibroblasts exposed to abnormal stiffness or cyclic strain become pro-fibrotic, secreting excessive ECM and reinforcing scars [251,252,253]. However, the Mechanobiology Paradox is that the same forces, when carefully applied, can guide constructive alignment. Low-intensity ultrasound, acoustic fields, and nanostructured scaffolds have been shown to promote angiogenesis, orient collagen, and improve MSC differentiation. Rather than being an obstacle, controlled mechanical input can be transformed into a lever for regeneration.

**Metabolic reprogramming.** The metabolic state of wounds is equally decisive. Chronic ulcers often display impaired mitochondrial function and an inflexible reliance on glycolysis, which fosters ROS accumulation and senescence [254,255]. The Metabolic Paradox is that metabolic rigidity, while destructive in situ, can become an entry point for intervention. NAD^+^ boosters, ketone esters, and targeted amino acid therapies are emerging as tools to reset redox balance, enhance mitochondrial resilience, and bias secretome composition toward pro-regenerative signaling. Coupling these approaches with MSC/EV therapies reframes metabolism from a barrier into a programmable axis of healing.

Mechanobiology and metabolism underscore that wounds are not static defects but dynamic systems shaped by forces and fuels. By aligning mechanical cues and reprogramming metabolic states, regenerative therapies may overcome traps that have resisted conventional interventions (Supplementary Table S20).

### 5.7. DMSO: The Vector Paradox

Dimethyl sulfoxide (DMSO) is one of the most paradoxical agents in regenerative medicine. Best known as a cryoprotectant indispensable for stem cell preservation, it has also been used in an anecdotal clinical context as a topical agent for wound healing, arthritis, and inflammatory conditions [256]. This dual identity positions DMSO at the boundary between laboratory routine and unorthodox therapy, making it an instructive case study of how ancillary agents can influence regenerative outcomes.

The trap lies in its role as a cryoprotectant. At the concentrations typically used for cryostorage (5–10% *v*/*v*), DMSO is cytotoxic, compromising MSC viability and destabilizing EVs upon transplantation if not adequately washed out [257,258]. In clinical contexts, unregulated topical use may exacerbate tissue irritation, confound interpretation of outcomes, and undermine regulatory acceptance. Moreover, its ability to act as a molecular vector can become a liability when unintended substances, including contaminants or pro-inflammatory metabolites, are transported deep into tissues, amplifying toxicity.

DMSO also carries potential as a niche-supporting trick. At low, carefully titrated concentrations, DMSO exhibits antioxidant, anti-inflammatory, and membrane-permeabilizing properties [256]. These effects can reduce oxidative stress in wounds, enhance penetration of regenerative factors, and stabilize the microenvironment for transplanted MSCs or EVs. Its vector property can be harnessed therapeutically: by co-formulating DMSO with bioactive molecules or herbal compounds, it can deliver agents otherwise constrained by molecular size or polarity deep into wound tissue, thereby expanding the repertoire of regenerative adjuncts [259,260,261,262]. Preclinical reports suggest that DMSO may modulate ion fluxes and redox balance, thereby aligning with broader strategies of niche reprogramming [263].

However, results remain controversial. While some animal studies in acute spinal cord injury and ischemia–reperfusion models reported neuroprotective and anti-scarring effects when DMSO was administered early, other studies failed to demonstrate meaningful functional recovery, and in vitro data indicate dose-dependent toxicity to neurons and glia [264,265,266,267]. Clinical evidence is anecdotal at best, with no robust trials confirming benefit in wounds or CNS repair. This inconsistency highlights DMSO as both a trap and a research opportunity: promising as a niche modulator, but prone to failure without strict attention to dose, timing, and context.

Despite decades of informal use, robust clinical trials are lacking, and regulatory acceptance remains minimal. Nevertheless, DMSO illustrates a critical lesson: compounds long considered ancillary can emerge as active modulators of stem cell niches. It therefore serves as a cautionary tale and a conceptual bridge, demonstrating that the boundary between trap and trick is often dose-, context-, and regulation-dependent, and setting the stage for unconventional adjuncts explored in Section 4 (Supplementary Table S21).

## 6. Synthesis: From Traps, Paradoxes, and Tricks to Paradigm Shifts

The preceding sections have treated wound healing as a succession of obstacles and innovations—hostile niches, fragile therapies, and incremental tricks that buffer their loss. However, these insights point to something more profound: the failure of stem cell and exosome therapies is not stochastic, but patterned. Wounds expose systemic bottlenecks—metabolic, immune, microbial, bioelectric—that conventional delivery cannot circumvent. These bottlenecks, when recurrent across trials and models, function less as random pitfalls than as stress tests that reveal the hidden architecture of regeneration.

It is in this sense that traps, paradoxes, and tricks must be synthesized. Rather than cataloging isolated phenomena, this analysis demonstrates that regenerative inconsistency reflects the operation of network-level regulators that shape whether vesicle traffic and cell signals converge on repair or collapse into fibrosis. To move beyond descriptive biology, the field requires integrative frameworks that translate lessons of failure into predictive maps of outcome. Two such frameworks are proposed here: the Triad-Exosome Axis, which explains the recurrent instability of MSC/EV therapies as a systems failure model, and the Multi-Axis Regeneration Map, which extends this view across neuroimmune, metabolic, and temporal dimensions.

### 6.1. The Triad-Exosome Axis: A Systems Failure Model

At the heart of regenerative inconsistency lies the vesicle. Exosomes and other EVs are not autonomous therapeutics but mirrors of their systemic context. They faithfully reproduce the metabolic, immunological, and microbial tone of the environment from which they arise. This plasticity makes them attractive as regenerative surrogates, but it also explains why outcomes fluctuate across patients and models. What has been described as therapeutic “noise” is in fact a signature of a more profound network imbalance.

The Triad-Exosome Axis is proposed as a unifying framework. Three regulators—mitochondria, mast cells, and microbiota—form a triangular control system that dictates the functional polarity of EV cargo. Mitochondria impose the redox and metabolic code: oxidative stress or NAD^+^ depletion biases vesicles toward senescence-associated microRNAs, while metabolic flexibility enriches angiogenic and anti-inflammatory signals [268]. Mast cells act as sensors and amplifiers: under controlled activation, they release mediators that recruit repair, but when hyperactivated, they flood the niche with proteases and histamine that degrade MSCs and reprogram EVs toward inflammatory phenotypes [269,270,271]. Microbiota contribute a parallel stream of vesicles. In balanced states, these may reinforce barrier function; in dysbiosis or biofilm dominance, they compete with regenerative signals, exporting toxins or pro-fibrotic cues [103,272].

**Figure 2:**
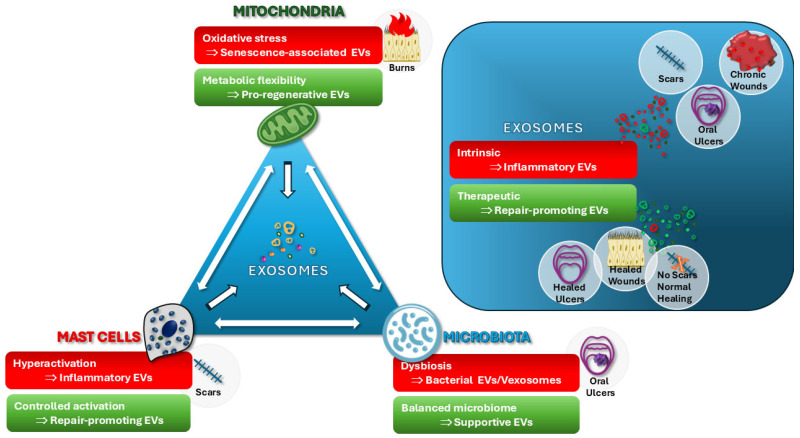
The Triad-Exosome Axis: A Systems Model of Regenerative Control and Failure. The triangular framework illustrates how three interconnected regulators: mitochondria, mast cells, and microbiota—collectively determine the polarity of exosome cargo and therapeutic outcomes. Each node displays both balanced (green) and dysregulated (red) states with EV profiles. In the balanced state, mitochondria generate pro-regenerative EVs through metabolic flexibility, mast cells produce repair-promoting EVs via controlled activation, and microbiota contribute supportive EVs from a balanced microbiome. Conversely, dysregulation produces vexosomes: mitochondrial oxidative stress generates senescence-associated EVs, mast cell hyperactivation releases inflammatory EVs, and dysbiotic microbiota secrete bacterial EVs, i.e., vexosomes. The central exosome pool represents the competitive balance between intrinsic vexosomes (red) and therapeutic repair-promoting EVs (green). Successful healing occurs when therapeutic EVs outnumber and functionally override vexosomes, as illustrated in the framed panel showing predominant green vesicles associated with healed wounds. Bidirectional arrows between nodes indicate crosstalk and mutual regulation, while unidirectional arrows toward the central exosomes demonstrate how each triad member influences EV cargo composition. Representative wound types are positioned according to their dominant triad dysfunction: burns (mitochondrial oxidative collapse), hypertrophic scars and keloids (mast cell hyperactivation), and oral ulcers (microbiota dysbiosis with vesicle interference). This systems model explains the inconsistency of MSC and exosome therapies as a consequence of triad imbalance rather than inherent therapeutic limitations, providing a predictive framework for context-aware regenerative interventions.

When the triad is dysregulated, exosomes become pathological vectors, propagating fibrosis, immune exhaustion, or microbial mimicry. When the triad is balanced, exosomes function as regenerative catalysts, coordinating angiogenesis, fibroblast remodeling, and epithelial closure [273]. The inconsistency of MSC/EV trials is therefore not primarily a reflection of product quality, but of triad state at the time of administration (Figure 2).

This systems view reframes the challenge: stem cell and exosome therapies cannot be evaluated apart from their ecological regulators. Success does not depend only on the dose or formulation of therapeutic vesicles, but on whether the mitochondrial, mast cell, and microbial nodes of the triad are aligned to receive and amplify their signals. In this sense, the Triad-Exosome Axis represents a foundational paradigm shift—transforming scattered trial failures into a predictive framework that fundamentally reframes why regenerative therapies succeed or fail. 

### 6.2. Predictive Matrix

If the Triad-Exosome Axis offers a systems failure model, its practical value lies in prediction. Clinical inconsistency across MSC and EV trials has often been ascribed to product variability. However, a closer view suggests that outcomes depend less on the therapy and more on the state of the triad at the time of intervention. Different wound types exhibit characteristic imbalances—mitochondrial collapse, mast cell hyperreactivity, or microbiota dysbiosis—which leave distinct signatures in the vesicles that circulate through the wound niche.

The predictive matrix below (Table 6) illustrates this principle. Each wound type can be aligned with a dominant dysfunction in the triad and the typical EV cargo it produces. Importantly, dominance does not imply exclusivity. A diabetic ulcer may be defined by mitochondrial collapse, but mast cell hyperactivation and microbial dysbiosis amplify the same trajectory. Likewise, a keloid scar is often driven by mast cell-fibroblast cross-talk; however, microbial vesicles and oxidative stress reinforce its pro-fibrotic signature. The triad functions as a coupled system, in which an imbalance at one node reverberates across the others. 

By linking wound type to triad dysfunction and EV profile, the matrix provides a scaffold for patient stratification. It reframes clinical “failures” not as evidence against MSCs or exosomes, but as consequences of unrecognized systemic biases. What appears as inconsistency is, in fact, a form of hidden order—a predictable pattern once the triad is taken as the operative unit. This perspective suggests that regenerative therapies will succeed not through universal dosing but through context-aware alignment with the triad state, supported by adjuncts that restore balance across its interconnected nodes.

### 6.3. Multi-Axis Regeneration Map: Beyond the Triad

The Triad-Exosome Axis provides a framework for interpreting inconsistency in regenerative outcomes, but it is not the whole picture. Wounds are not governed by three nodes alone; they are shaped by a constellation of interacting axes that extend beyond mitochondria, mast cells, and microbiota. Together, these axes form a multi-dimensional control map, where success or failure emerges not from a single defect but from the (mis)alignment of systems. Several axes extend and enrich this view:

**Neuroimmune axis.** Neuropeptides such as CGRP and Substance P, along with vagal pathways, dictate whether immune tone is permissive or hostile to transplanted products. Their misalignment explains accelerated clearance and the persistence of neurogenic inflammation in chronic wounds.

**Bioelectric axis.** Endogenous electrical polarity organizes electrotaxis and closure; when collapsed, it produces chaotic migration. External stimulation or conductive scaffolds can reset this axis, reframing electrical disorganization as a programmable variable.

**Circadian axis.** Oscillations in immunity, perfusion, and proliferation generate temporal windows of opportunity. Ignoring them produces temporal blindness; synchronizing therapy with circadian rhythms converts time into a therapeutic lever.

**Metabolic axis.** Chronic wounds are often locked in a glycolytic bias with mitochondrial dysfunction. Resetting metabolic flexibility through NAD^+^ boosters, ketone esters, or amino acid cues reframes rigidity as an entry point for reprogramming.

**Hematologic axis**. Erythrocytes, long regarded as inert, may under specific conditions exhibit plasticity, mediated through surface glycoproteins and vesicle exchange. This speculative axis raises the possibility that the circulatory system functions as a latent regenerative organ.

**Pathobiota–wound axis.** Dysbiosis transforms commensals into pathobiota, exporting vesicles that act as pathological antagonists to regeneration. These vesicles—here termed vexosomes—carry toxins, quorum-sensing molecules, and fibrosis-associated cargo that compete with therapeutic EVs for cellular uptake and immune signaling. The trap lies in assuming microbial activity is background noise; the paradox is that the same communities can reinforce repair in balance or sabotage it in dysbiosis. The trick is to bias this vesicle ecology, through microbiome engineering, probiotics, or cross-kingdom vesicle modulation, so that regenerative exosomes are amplified while vexosomes are neutralized.

**Organ–wound axes.** Wounds are not isolated lesions but reflections of systemic states. The gut–skin axis links microbial dysbiosis to systemic inflammation that undermines wound repair; the oral-systemic axis connects periodontal pathogens with delayed healing and a fibrotic bias; the neuroimmune axis ties stress or neuropathy to an altered inflammatory tone. These relationships illustrate that a wound is often less a local accident than a sentinel readout of broader organ dysfunction. The paradox here is that systemic disease can sabotage local repair. Still, conversely, interventions at distant sites, such as gut microbiome restoration, oral infection control, or vagal modulation, can unlock regenerative potential in wounds that are otherwise resistant to local therapy.

These axes do not act in isolation. Circadian disruption can exacerbate oxidative stress, which destabilizes the cargo of mitochondrial vesicles. Microbial vexosomes can potentiate mast cell hyperactivation, amplifying fibrosis. Loss of bioelectric polarity accelerates microbial colonization and immune mis-education. The wound niche is thus a node of convergence, where multiple axes intersect to determine whether healing proceeds toward regeneration or collapses into chronicity.

**Figure 3:**
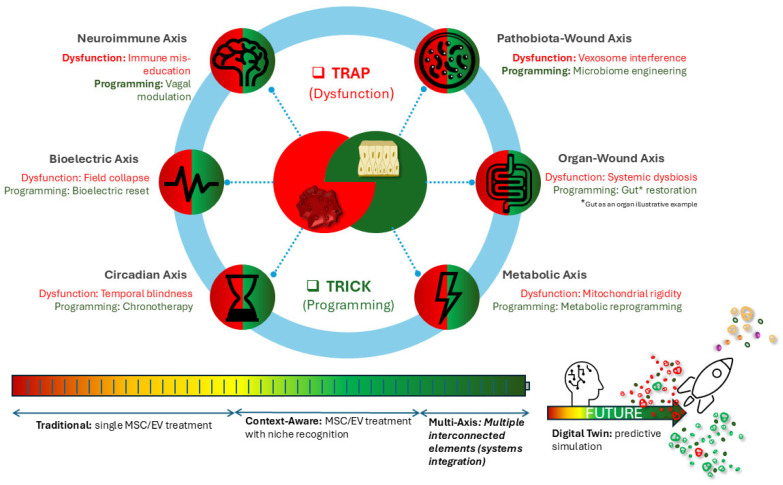
Multi-Axis Regeneration Map: From Single Interventions to Systems Programming. The framework illustrates six interconnected axes that govern wound healing outcomes: neuroimmune, bioelectric, circadian, metabolic, pathobiota–wound, and organ–wound axes. Each axis exhibits both dysfunctional states (red, “traps”) that undermine regenerative therapies and programmable interventions (green, “tricks”) that can be therapeutically targeted. The central yin-yang configuration illustrates the dynamic balance between pathological (red) and regenerative (green) states, showing how wound outcomes depend on the equilibrium between competing biological forces influenced by the surrounding axes. The blue connecting ring highlights the interconnected nature and mutual influence among all axes, illustrating that intervention on one axis generates ripple effects throughout the entire system. The red and green coloring directly corresponds to the trap (dysfunction) and trick (programming) states of each axis, emphasizing that wounds exist at the intersection of these opposing forces. Dotted lines indicate specific cross-axis influences, while the ring represents the broader systems-level integration. The therapeutic evolution spectrum (bottom) illustrates progression from traditional single MSC/EV treatments to context-aware niche recognition, and ultimately to multi-axis systems integration (the approach advocated in this review), with digital twin-guided predictive simulation representing the future frontier. The color gradient from red to green reflects increasing therapeutic sophistication and success probability. This framework reframes wound healing from a localized tissue defect into a systems-level phenomenon requiring coordinated intervention across multiple biological dimensions. Unlike isolated single-axis approaches that often fail due to compensatory dysfunction in other axes, multi-axis programming addresses the interconnected nature of regenerative control, transforming traps into programmable therapeutic levers for context-aware, precision wound care.

The Multi-Axis Regeneration Map represents a dynamic theoretical advance that enables systematic integration of previously disconnected biological systems. It invites researchers to chart how interventions along one axis ripple across others, and how combinations, such as bioelectric reset plus circadian alignment, or senolytics plus microbiome modulation, may achieve what single interventions cannot. Where the Triad-Exosome Axis diagnoses failure, the Multi-Axis Map sketches a future of context-aware, combinatorial, and programmable regeneration (Figure 3). To anchor the Triad-Exosome Axis and the Multi-Axis Map in measurable reality, we map exemplar wound types to a minimal biomarker set and state explicit, falsifiable predictions that can be tested prospectively (Table 7). Detailed measurement panels are provided in Supplementary Note S1, and Tables S29 and S30a–d, with dataset pointers in the accompanying comments below the tables. An extended overview of exploratory or integrative modalities with anecdotal or early observational support is provided in Supplementary Table S24, highlighting areas where formal mechanistic and clinical validation are warranted.

### 6.4. From Failure Lessons to Paradigm Shifts

This synthesis highlights a broader point: paradigm shifts in regenerative medicine will not emerge from perfecting single interventions, but from reframing traps as paradoxes and programming them into tricks. The Triad-Exosome Axis provides a failure model, while the Multi-Axis Map offers a blueprint for interventions. Together, they position wounds not as pathological accidents but as stress tests of regenerative capacity—unique laboratories where the limits of cell, vesicle, and systemic biology are exposed. By decoding these failures, the field is moving toward a new regenerative paradigm: one that is context-aware, programmable, and systems-integrated for promoting healing.

## 7. Conclusions and Outlook: Programming Regeneration from Traps to Paradigms 

This review deliberately adopts the metaphorical framework of ‘traps and tricks’ to align with the Special Issue theme while providing a systematic reconceptualization of failures in regenerative medicine. ‘Traps’ represent not merely obstacles but predictable system vulnerabilities that can be decoded and addressed. ‘Tricks’ are not simple workarounds, but strategic interventions that transform systemic liabilities into therapeutic opportunities. This framework moves beyond the traditional ‘challenges and solutions’ paradigm by emphasizing the dynamic, programmable nature of regenerative systems, where apparent barriers become entry points for intervention once their underlying logic is understood.

The trajectory of stem cell and exosome therapies in wound healing has often been narrated as a cycle of promise and disappointment. However, viewed through the lens of traps, paradoxes, and tricks, a different pattern emerges. Failures cease to be endpoints; they become data-rich signals that expose the operating principles of regeneration. Every hostile microenvironment, every paradoxical scarring response, and every misleading vesicle population has provided clues about how wounds are regulated, and how therapies must be reprogrammed to succeed.

The novel theoretical frameworks developed here—the Triad-Exosome Axis, the Predictive Matrix, and the Multi-Axis Regeneration Map—establish a new conceptual foundation for regenerative medicine. Combined, they shift the field from a product-centric focus toward a context-aware model of healing, in which the success of MSCs and EVs is inseparable from the metabolic, microbial, immune, electrical, and systemic axes that govern their fate. This reconceptualization transforms wounds from frustrating anomalies into stress tests of regenerative capacity, laboratories where the limitations of conventional approaches are made visible.

The next phase of regenerative medicine will demand programmability. Cells and vesicles must be deployed not as static payloads but as responsive agents, aligned with circadian windows, embedded in engineered niches, protected from vexosomes, and synchronized with systemic states across organ–wound axes. Such a shift will not eliminate traps; it will harness them, turning liabilities into design variables. Emerging platforms demonstrate that regenerative medicine is no longer a conceptual but a tangible reality. Bioelectric-hydrogel hybrids merge conductive polymers, such as graphene, PEDOT:PSS, or polypyrrole, with bioactive scaffolds, enabling spatial control of electrical cues that synchronize cell migration, angiogenesis, and exosome release. Smart exosome–hydrogel systems utilize pH-, ROS-, or enzyme-responsive hydrogels to deliver engineered vesicles only when wound microenvironments reach permissive thresholds, thereby converting biochemical noise into a timed regenerative output. Circadian-sensor platforms integrate wearable biosensors with feedback-controlled release modules, aligning regenerative therapy with circadian immune and metabolic rhythms. These examples embody the shift from static interventions to adaptive, feedback-driven, and context-aware therapeutics, defining the next horizon of programmable regeneration.

In this light, the outlook for stem cell and exosome therapies is not defined by whether they “work” in an absolute sense, but by how the field learns to work with them to navigate the challenges, decode the paradoxes, and transform them into programmable levers. The paradigm of regenerative wound therapy is no longer a binary of success or failure. It is a continuum of contextual control, where healing becomes a process that can be guided, tuned, and ultimately programmed.

## Figures and Tables

**Table 1:** Glossary of Key Concepts.

Key Concept	The Concept Description
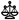 Infection-Control Paradox	Antimicrobial dressings and antiseptics essential for infection suppression (e.g., silver, iodine, chlorhexidine) paradoxically destabilize MSCs and EVs, impair host cell proliferation, and degrade vesicle cargo. What protects against microbes can simultaneously sabotage regeneration.
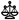 Dosing Paradox	The assumption that higher doses of MSCs/EVs yield better outcomes; paradoxically, large bolus doses may accelerate washout, metabolic stress, immune clearance, and receptor desensitization, leading to reduced efficacy.
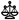 Scarring Paradox	The dual role of MSCs and EVs in modulating fibrosis: under supportive conditions they suppress scarring, but in dysregulated niches they may inadvertently reinforce fibrotic signaling and hypertrophic scarring.
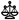 Immune Double-Edged Sword	The dual role of the immune system in stem cell and exosome therapy: clearance by neutrophils, macrophages, and complement reduces therapeutic persistence, yet controlled immune modulation is also essential for regenerative benefit. The same pathways that enable healing can simultaneously accelerate therapeutic loss or create tumor-permissive environments.
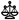 Neuroimmune Scarring	The maladaptive interaction of neural and immune signals that promotes persistent fibrotic or glial scars. In wounds, disrupted neuropeptides (CGRP, Substance P) and impaired vagal tone drive hyper-clearance of MSCs/EVs and reinforce fibrosis. In the CNS (central nervous system), similar mis-education underlies glial scar formation, walling off axonal regrowth, and preventing full spinal cord and nerve regeneration.
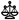 Vector Paradox	The dual role of DMSO as a molecular vector: it can inadvertently carry toxins or contaminants deep into tissue, amplifying harm, yet can also be harnessed to deliver therapeutic molecules or adjuncts (e.g., antioxidants, herbal bioactives) across biological barriers to support regeneration.
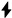 Bioelectric Reset	The restoration of endogenous wound polarity (typically 40–200 mV/mm) using external electrical stimulation or conductive biomaterials. Loss of polarity creates electrical disorganization, a hidden trap that disrupts electrotaxis and regenerative integration. Resetting the bioelectric field transforms this liability into a programmable trick that guides cell migration, enhances MSC survival, and optimizes EV cargo release.
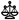 Preconditioning Paradox	Stressors that typically destroy MSCs and EVs in vivo (hypoxia, oxidative bursts, cytokine exposure) can, when applied in controlled in vitro settings, enhance resilience and enrich therapeutic cargo. What is destructive in the wound becomes constructive in preparation, turning stress into a tool for regenerative programming.
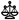 Exosome Paradox	The same property that makes exosomes therapeutically powerful—their ability to mirror the physiological state of their parent cells—also makes them vehicles of pathology. Under stress or dysbiosis, they propagate senescence, fibrosis, or microbial mimicry. Filtering and engineering are therefore required to convert their double agency into a reliable regenerative tool.
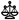 Stratification Paradox	The more heterogeneous the patient cohort, the less effective regenerative therapies appear to be in trials; yet when therapies are precisely matched to patient-specific trap profiles, their benefits are amplified. Failed RCTs often reflect a misalignment between therapy and context, rather than the intrinsic inefficacy of MSCs or EVs.
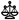 Herbal Paradox	Natural compounds are often dismissed as anecdotal or low-tech, yet they intervene precisely at systemic choke points—oxidative stress, fibrosis, microbial competition, and EV signaling. When standardized and deliberately deployed, they act as niche buffers and as programmable regulators of vesicle cargo and regenerative timing.
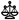 Systemic Synergy Paradox (Herbal Systems)	Herbal preparations should not be reduced to single “active” compounds. What appears unrefined—a whole herb or extract—may in fact deliver superior regenerative programming through its internal network of constituents. Their regenerative effects often arise from the coordinated interactions of multiple constituents, creating layered effects: one compound buffers toxicity, another stabilizes delivery, and others act as catalytic triggers of broader cascades. This internal harmony generates outcomes greater than the sum of parts, reframing herbs as complex systems that can empower stem cell and exosome therapies rather than mere adjuncts.
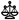 Circadian Paradox (Temporal Stratification)	The same circadian rhythms that accelerate clearance or resistance when ignored can amplify regenerative benefit when harnessed. The wound response is not constant throughout the day but oscillates in relation to immune tone, vascular perfusion, and proliferative cycles. Treating time as a programmable variable reframes temporal blindness from a trap into a therapeutic lever.
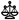 Predictive Simulation Paradox	The very complexity and heterogeneity that make wound healing unpredictable can, when captured in digital twins or computational models, become a source of precision. What appears as noise in clinical practice is reframed as actionable data, allowing traps to be anticipated and regenerative therapies to be personalized in silico before deployment.
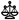 Erythrocyte Plasticity Paradox (Speculative)	Red blood cells, long considered terminally differentiated and inert, may, under certain conditions, display latent regenerative potential. What appears as maximal specialization for oxygen transport may conceal hidden plasticity, mediated by surface glycoproteins and vesicle exchange. This reframes erythrocytes from passive carriers to abundant systemic reservoirs that could be co-opted for regenerative programming.
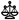 Mechanobiology Paradox	Mechanical forces that drive fibrosis when uncontrolled can, under precise application, guide regenerative alignment and angiogenesis.
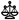 Metabolic Paradox	Metabolic rigidity that locks wounds into a state of chronic inflammation can, when reprogrammed with targeted metabolic cues, become a lever for regenerative signaling.
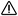 Vexosomes	Proposed definition: Pathological EVs generated by microbes (bacteria, fungi, viruses) or host cells under conditions of dysbiosis, inflammation, or immune-microbial cross-talk. Unlike regenerative exosomes, vexosomes carry toxic, fibrotic, or pro-senescent cargo that propagate ecosystem failure locally and systemically. They represent the antagonistic counterpart to therapeutic EVs, and their presence defines the critical component of the pathobiota–wound axis.
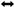 Triad-Exosome Axis	A systems-level framework linking mitochondria, mast cells, and microbiota to the generation of pathological EVs that derail regeneration.
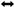 Multi-Axis Regeneration Map	An integrative framework mapping how wound healing is influenced by systemic axes (gut–skin, oral-systemic, neuroimmune), reframing local wounds as systemic phenomena.

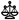
 Paradox—double-edged biological mechanisms; 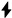 Bioelectric concept—endogenous currents, polarity, wound bioelectricity; 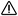 Caution—emerging concept with potential translational or safety implications; 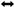 Systems-level framework—conceptual axes and models.

**Table 2:** Trap-Trick Capsule: Infection-Control Paradox. Core Insight: What protects against microbes can inadvertently sabotage MSC/EV-mediated healing.

Trap	Trick
Antimicrobials (e.g., silver, iodine, chlorhexidine) reduce bioburden but simultaneously damage MSCs/EVs and destabilize regenerative signals.	Restrict to early infection control, then transition to cell-friendly carriers or protective scaffolds to preserve regenerative viability.

**Table 3:** Representative evidence landscape for selected modalities in stem cell- and exosome-based wound therapy [23,51,52,53,54,55,56,57,58,59,60,61,62,63,64,65,66,67,68,69,70,71,72,73,74,75,76,77,78,79,80,81].

Modality/Concept	Mechanistic Focus	Representative Evidence	Evidence Level	Comment
Silver/Iodine dressings	Oxidative stress; preclinical evidence of EV and MSC membrane perturbation; antimicrobial release.	Silver: Mixed closure rates across DFU/VLU RCTs: benefits more consistent in DFU. Iodine: Limited DFU evidence; not routinely recommended; Some RCTs show benefit in VLU, but heterogeneity and bias limit certainty	A–B (clinical outcomes) C (mechanisms)	Silver: Efficacy dose- and formulation-dependent; in vitro cytotoxicity often observed above ~10 ppm Ag^+^, with thresholds varying by cell type and medium. Iodine: May reduce bacterial burden; closure benefit inconsistent across trials
HOCl antiseptics	Reactive-oxygen modulation; broad antimicrobial with low toxicity	In vitro MSC viability preserved; small clinical cohorts show safety and wound improvement	B	Well-tolerated; low cytotoxicity compared to silver/iodine
Medical-grade honey	Osmotic/enzymatic antimicrobial; mild pro-angiogenic signaling	Meta-analyses and RCTs in DFU and burns show improved healing and infection control	A	Consistent safety; modest acceleration of granulation tissue formation; widely accepted as adjunct therapy
Electrical stimulation	Field-guided cell migration and angiogenesis	Controlled animal and small human trials show accelerated healing	B	Promising but heterogeneous parameters; standardization needed
Dosing paradox/receptor desensitization	Saturation of trophic signaling and EV uptake pathways	Preclinical cytokine/EV overload models show reduced efficacy at high bolus doses;	C–D	Large bolus doses may accelerate washout, metabolic stress, immune clearance, and receptor desensitization, paradoxically reducing efficacy. Hypothesis-generating; human validation lacking. Supports exploration of fractionated or sustained-release dosing regimens for MSC/EV therapies.
MSC-derived extracellular vesicles (MSC-EVs)	Immunomodulation; angiogenesis; matrix remodeling	Early RCTs and numerous animal studies show promise in wound healing and tissue repair	A–B	Standardization and potency assays in progress; manufacturing and dosing protocols still under development.
Senolytics (quercetin, verteporfin)	Targeting senescent fibroblasts; antifibrotic modulation	Animal scar models; pilot human data suggest reduced fibrosis and improved remodeling	B–C	Experimental adjunct; dosing and delivery strategies under study; long-term safety and efficacy not yet established.
Botanical extracts (e.g., plantain, curcumin)	Anti-inflammatory and antioxidant signaling	Mostly in vitro and rodent wound models; limited controlled human data	C–D	Variability in extract preparation, dosing, and bioavailability; need for standardized formulations and controlled trials.
Photobiomodulation/optogenetic hybrids	Light-triggered bioelectric activation of repair cascades, angiogenesis, and cell migration.	Early preclinical and feasibility trials; limited translational data	C–D	Horizon technology; safety/parameter window undefined; requires rigorous standardization before clinical adoption

**Table 4:** Trap-Trick Capsule: Dosing Paradox. Core Insight: More is not better; smart delivery outperforms high-dose bolus strategies.

Trap	Trick
Large bolus doses increase washout, metabolic stress, apoptosis, and receptor desensitization, reducing efficacy.	Fractionated, niche-aware delivery (hydrogels, patches, repeated microvolumes) enhances retention and functional exposure.

**Table 5:** Trap-Trick Capsule: Scarring Paradox. Core insight: The same regenerative cues that suppress fibrosis in a stabilized wound niche may reinforce scarring when delivered into an inflamed, dysregulated, or mechanically stressed niche; context, not the product, determines directionality.

Trap	Trick
Under dysregulated inflammation and mechanical stress, MSCs/EVs may unintentionally amplify profibrotic signaling, worsening hypertrophic or keloid scarring.	Stabilize the wound niche (timing, inflammation control, ECM support) to shift MSC/EV signaling toward antifibrotic pathways rather than fibrosis reinforcement.

**Table 6:** Predictive Matrix Linking Wound Type, Triad Dysfunction, and EV Cargo. The triad members are always presented in the same order for consistency (Mitochondria, Mast cells, Microbiota); primary triad driver of dysfunction in bold; secondary contributions in plain text.

Wound Type	Triad Dysfunction (Mitochondria—Mast Cells—Microbiota)	Typical Pathological EV Cargo	Implication for MSC/EV Therapy
Burns	**Mitochondria:** severe ROS surge, loss of redox balance; Mast cells: acute degranulation; Microbiota: secondary colonization	Oxidized proteins, mtDNA fragments, pro-inflammatory miRNAs	Poor survival of transplanted MSCs; EVs degraded or overwhelmed by oxidative stress
Chronic wounds (diabetic, venous, pressure)	Mitochondria: metabolic stress; Mast cells: chronic hyperactivation; **Microbiota:** dysbiosis, biofilm EVs	Bacterial EVs carrying toxins; EVs with pro-inflammatory cytokine cargo	MSC/EV therapy neutralized by microbial competition; persistence of inflammation
Grafting failures	**Mitochondria:** ischemia-driven energy collapse; Mast cells: local degranulation at graft margins; Microbiota: opportunistic colonization	EVs enriched in DAMPs and pro-fibrotic factors	Accelerated graft necrosis, fibrosis, poor integration
Oral ulcers	Mitochondria: variable stress; Mast cells: neuropeptide-driven hyperreactivity; **Microbiota**: complex dysbiosis with bacterial EV interference	Microbial EVs; neuropeptide-influenced EV signaling	MSC/EV therapies washed out; EVs cleared rapidly, poor functional integration
Keloids/hypertrophic scars	Mitochondria: moderate oxidative imbalance; **Mast cells**: fibroblast-mast cell axis dysfunction; Microbiota: dysbiotic contribution	Fibrosis-associated EVs (TGF-β, collagen cross-linking enzymes, pro-fibrotic miRNAs)	MSC/EV therapy risks reinforcing fibrosis unless combined with anti-fibrotics

**Table 7:** Axis anchoring with exemplar wounds and a minimal biomarker set (clinical-ready).

Exemplar Wound	Dominant Axis (Triad-Exosome)	3–5 Pragmatic Biomarkers (baseline)	Interpretation of Axis State	Falsifiable Prediction
DFU (ischemic/infected)	Metabolic ↔ Microbiome	tcPO_2_; lactate; pH; biofilm score; EV miR-146a; optional endotoxin load	Indicates metabolic rigidity (high lactate, low pH) plus microbial-EV interference (endotoxin, low miR-146a) suppressing regenerative EV signaling	If lactate + endotoxin elevated, standard care + low-toxicity binder/phyto-detox adjunct (see Supplement Table S5) will reduce endotoxin and increase EV miR-146a within 2–4 weeks, correlating with faster granulation.
VLU (inflammatory)	Immune ↔ Metabolic	CRP/IL-6; mast-cell tryptase; MMP-9/TIMP-1; TEWL; EV miR-21	Reflects protease-dominant, mast-cell–active inflammatory state with pro-fibrotic EV cargo (miR-21), impairing angiogenesis and epithelialization	If MMP-9/TIMP-1 ratio and tryptase are elevated, MC-modulating + matrix-stabilizing therapy will normalize the ratio and improve granulation by week 4.
PU (biofilm-heavy)	Microbiome ↔ Immune	Quantitative bioburden; QS molecules (AHL proxy); HOCl challenge response; EV endotoxin assay	Indicates microbial quorum-signaling dominance, high biofilm resistance, and endotoxin-rich EV milieu that blocks MSC/EV uptake	If QS signal high and HOCl kill-curve poor, intensified debridement + staged antisepsis will reduce QS signal and maintain EV viability.
Burns/graft take	Immune ↔ Mechanobiology	IL-8; VEGF; wound potential (mV/mm); elasticity (cutometer)	Reflects overwhelming neutrophilic inflammation (IL-8), disrupted angiogenic balance, and collapsed endogenous electrical fields predictive of graft failure	If wound potential is low and IL-8 high, validated bioelectric support will raise potential magnitude and correlate with improved graft take
Keloid/HTS	Immune ↔ Metabolic (fibrotic bias)	TGF-β1; CTGF; collagen I/III ratio; EV miR-29; mast-cell mediators	Indicates fibrotic axis dominance with TGF-β1–driven metabolic rigidity and depleted anti-fibrotic EV miRNAs (miR-29)	If TGF-β1 high and miR-29 low, antifibrotic trick (senolytic/verteporfin or EV-miR-29 enrich) will normalize collagen I/III trajectory over 6–12 weeks.

Abbreviations: DFU—diabetic foot ulcer; VLU—venous leg ulcer; PU—pressure ulcer; HTS—hypertrophic scar; tcPO_2_—transcutaneous oxygen; TEWL—transepidermal water loss. To anchor the Triad-Exosome Axis and the Multi-Axis Map in measurable reality, exemplar wound types are mapped to minimal biomarker sets with explicit, falsifiable predictions. Optional metabolic-detox markers (endotoxin, trace metals) and adjunct hypotheses are detailed in Supplementary Tables S29 and S30.

## Data Availability

No new data were created or analyzed in this study. Data sharing is not applicable to this article.

## Supplementary Materials

The following supporting information can be downloaded at: https://www.mdpi.com/article/10.3390/biomedicines13123030/s1, Supplement S1: Atlas of Trap-Trick Capsules; Supplementary Table S1: Trap-Trick Capsule: Microenvironmental Hostility; Supplementary Table S2: Trap-Trick Capsule: Infection Control vs. Cytotoxicity. Key Paradox: Infection-Control Paradox—What protects against microbes simultaneously sabotages regeneration; Supplementary Table S3: Trap-Trick Capsule: Dosing and Delivery Pitfalls. Key Paradox: Dosing Paradox—Higher doses increase washout, stress, and clearance, leading to paradoxical loss of efficacy; Supplementary Table S4: Trap-Trick Encapsule: Fibrosis and Scarring Bias. Key Paradox: Scarring Paradox—MSCs/EVs may suppress or reinforce fibrosis depending on niche context; Supplementary Table S5: Trap-Trick Capsule: Immune Clearance and Neuroimmune Crosstalk. Key Paradox: Immune Double-Edged Sword—The same pathways that enable healing also accelerate therapeutic loss; Supplementary Table S6: Trap-Trick Capsule: Safety, Regulatory, and Economic Concerns; Supplementary Table S7: Trap-Trick Capsule: Burns; Supplementary Table S8: Trap-Trick Capsule: Chronic Wounds. Key Paradoxes: Dosing Paradox + Reverse Scarring Paradox—Higher doses worsen clearance; failure to mount adequate fibrotic closure leads to persistent open wounds; Supplementary Table S9: Trap-Trick Capsule: Grafting Strategies. Key Paradox: Scarring Paradox—MSCs/EVs accelerate closure but may reinforce fibrosis in dysregulated niches; Supplementary Table S10: Trap-Trick Capsule: Oral Ulcers. Key Paradoxes: Dosing Paradox + Microbiota-Exosome Axis—High doses accelerate clearance; microbial EVs compete with therapeutic signals; Supplementary Table S11: Trap-Trick Capsule: Preconditioning and Biomaterial Carriers. Key Paradox: Preconditioning Paradox—Stressors destructive in vivo become constructive preparation tools in vitro; Supplementary Table S12: Trap-Trick Capsule: Exosome Engineering and Pathology Filtration. Key Paradox: Exosome Paradox—The same property that makes EVs therapeutic also makes them vehicles of pathology; Supplementary Table S13: Trap-Trick Capsule: Anti-Fibrotic and Senolytic Combinations. Key Paradox: Scarring Paradox—MSCs/EVs may suppress or reinforce fibrosis depending on niche context; Supplementary Table S14: Trap-Trick Capsule: Patient Stratification and Personalization. Key Paradox: Stratification Paradox—The more heterogeneous the cohort, the less effective uniform therapy appears; Supplementary Table S15: Trap-Trick Capsule: Bioelectric Reset; Supplementary Table S16: Trap-Trick Capsule: Herbal Paradox. Key Paradox: Herbal Paradox—Natural compounds dismissed as low-tech intervene at precise systemic choke points; Supplementary Table S17: Trap-Trick Capsule: Circadian Synchronization. Key Paradox: Circadian Paradox—Rhythms that undermine therapy when ignored amplify it when harnessed; Supplementary Table S18: Trap-Trick Capsule: Digital Twins and Predictive Simulation. Key Paradox: Predictive Simulation Paradox—The Complexity that makes wounds unpredictable becomes a source of precision when modeled; Supplementary Table S19: Trap-Trick Capsule: Erythrocyte Plasticity. Key Paradox: Erythrocyte Plasticity Paradox—Cells designed for maximal specialization may conceal maximal plasticity; Supplementary Table S20: Trap-Trick Capsule: Mechanobiology and Metabolic Horizons. Key Paradoxes: Mechanobiology + Metabolic Paradoxes—Destructive forces and metabolic rigidity become therapeutic levers when controlled; Supplementary Table S21: Trap-Trick Capsule: DMSO. Key Paradox: Vector Paradox—DMSO can carry toxins deep into tissue or be harnessed to deliver therapeutic agents; Supplementary Table S22: Comparative clinical and translational attributes of mesenchymal stem cell (MSC) and exosome-based therapies in wound healing. The table highlights mechanistic distinctions, safety considerations, manufacturing feasibility, regulatory pathways, and therapeutic contexts. Exosome-based products currently demonstrate greater near-term potential for scalable and standardized clinical translation, whereas MSCs remain essential for complex tissue remodeling and as cellular biofactories for regenerative vesicle production; Supplementary Table S23: Regulatory and Safety Overview of Key Modalities Referenced in the Manuscript. Purpose: To summarize regulatory status, evidence maturity, and known safety considerations of selected agents and interventions mentioned in the perspective review. The table is provided for transparency and does not imply clinical endorsement; Supplementary Table S24: Clinical Landscape of MSC and MSC-Derived EV Trials in Wound Healing (Scoping Summary) [95,160,274,275,276,277,278,279,280,281,282,283,284,285,286,287]. Purpose: To anchor the traps–paradoxes–tricks framework in representative clinical evidence. Note: Values reflect key published trials; not a systematic extraction. PRP- Platelet-Rich Plasma; Supplementary Horizon Table S25: Emerging Frontiers in Regenerative Wound Therapy. Purpose: To summarize the conceptual and preclinical status of themes discussed in Section 5 (Bioelectric Reset, Herbal Paradox, Erythrocyte Plasticity, Vector Paradox), and related constructs in Section 6; Supplementary Table S26: Integrative and Emerging Adjuncts Potentially Influencing Wound Repair—Hypothesis-Generating Evidence. Purpose: To collate integrative or non-standard modalities that have reported favorable outcomes in limited or practice-based contexts. Inclusion does not imply endorsement or clinical validation; the table identifies hybrid or ecosystem-level interventions meriting systematic investigation; Supplementary Table S27: Reference Ranges, simplified. Note: Measuring “wound potential” in mV/mm is not a current clinical practice. It is a research technique. Diagnosis and prognosis of chronic wounds rely on standardized clinical assessments (perfusion, infection, size, etiology). EF -Electric Field; Supplementary Table S28: Clinical application of Electrical Stimulation (ES) for wound healing; Supplementary Note S1: Prioritized Trial Agenda for Next-Generation MSC/EV-Based Wound Therapies. Disclaimer: This agenda outlines research hypotheses, candidate comparators, and mechanistic endpoints for future controlled trials. It is not intended as clinical guidance or practice direction; Supplementary Table S29: Clinical Measurement Panels for Triad/Axis Assignment of wounds to specific axis states within the Triad-Exosome framework. Core Panel (Point-of-Care, 15-min assessment). *Optional metabolic-detox panel relevant to hybrid detox–nutritional interventions (see Supplementary Table S5); Supplementary Table S30a. Specialized panel for extracellular vesicle analysis to assess regenerative capacity and pathological signatures; Supplementary Table S30b. Immune/Fibrosis Panel (Laboratory assessment); Supplementary Table S30c: Microbiome Panel (Specialized assessment); Supplementary Table S30d: Mechanobiology Panel (Biomechanical assessment); Supplementary Table S30e: Sampling Timeline and Decision Points.

## Abbreviations

The following abbreviations are used in this manuscript:

CNS	Central Nervous System
MSCs	Mesenchymal Stem Cells
EVs	Extracellular Vesicles
ROS	Reactive Oxygen Species
ECM	Extracellular Matrix
DFU	Diabetic Foot Ulcers
VLU	Venous Leg Ulcers
HOCl	Hypochlorous Acid
DAMPs	Damage-Associated Molecular Patterns
SA-EVs	Senescence-Associated EVs
TGF-β	Transforming Growth Factor-β
CTGF	Connective Tissue Growth Factor
IDO	Indoleamine 2,3-dioxygenase
FDA	U.S. Food and Drug Administration
EMA	European Medicines Agency
ATMPs	Advanced Therapy Medicinal Products
MISEV	Minimal Information for Studies of Extracellular Vesicles
CGRP	Calcitonin Gene-Related Peptide
RCTs	Randomized Controlled Trials
GPI	Glycosylphosphatidylinositol
EV-LPS/EV-Endotoxin	Lipopolysaccharide Present on EVs
HVPC	High-Voltage Pulsed Current
IL-6, IL-8, IL-10	Interleukin-6/-8/-10
LAL	Limulus Amebocyte Lysate (Endotoxin Assay)
LPS	Lipopolysaccharide
MMP−9	Matrix Metalloproteinase−9
NTA	Nanoparticle Tracking Analysis
POSAS	Patient And Observer Scar Assessment Scale
VAS (Pain VAS)	Visual Analog Scale For Pain
