# Supplementary material for: Stem Cell and Exosome Therapy in Wound Healing: Traps, Paradoxes, and Tricks Transforming Paradigms
Source: Biomedicines. 2025 Dec 10;13(12):3030. doi: 10.3390/biomedicines13123030 (PMC12730715; doi:10.3390/biomedicines13123030)
Supplement: Supplementary file 1 [file biomedicines-13-03030-s001.zip › biomedicines-3924200-supplementary.pdf]

List of Supplementary Materials

Supplement S1. Atlas of Trap-Trick Capsules

This Supplement provides the complete set of Trap-Trick Capsules (Tables 1–21), which have been relocated from the main text for clarity and compactness. Traps represent obstacles; Tricks represent strategic solutions that leverage understanding of these obstacles. Each capsule distills a recurrent paradox in regenerative medicine - the trap - and its corresponding counter-strategy - the trick.

While the main text integrates these principles narratively within broader conceptual sections, the Atlas offers a structured, at-a-glance summary of the most representative mechanistic, translational, and systemic examples. Capsules are grouped by domain, spanning microenvironmental, immune, bioelectric, metabolic, and digital horizons, as well as clinical and adjunctive contexts.

The goal of this Supplement is to provide a concise, cross-referenced overview of all regenerative traps and tricks described in the main text. These capsules form a supplementary roadmap for researchers and clinicians aiming to identify where regenerative interventions most often fail (traps) and how they may be rationally redesigned (tricks).

Disclaimer: The Trap-Trick Capsules compiled in this Atlas are conceptual research frameworks intended to illustrate mechanistic hypotheses and translational directions. They are not clinical practice guidelines and should not be applied in patient care outside of controlled research protocols.

Supplementary Table S1. Trap-Trick Capsule: Microenvironmental Hostility

Traps	Corresponding Tricks
Necrosis, vascular thrombosis, and high inflammation drive a hostile microenvironment for MSCs/EVs	Encapsulation in protective biomaterials (HA, collagen scaffolds)
Natural bias toward fibrosis leads to hypertrophic scarring and contractures	Anti-fibrotic adjuvants (quercetin, YAP/TAZ inhibitors, senolytics)

Supplementary Table S2. Trap-Trick Capsule: Infection Control vs. Cytotoxicity. Key Paradox: Infection-Control Paradox - What protects against microbes simultaneously sabotages regeneration.

Traps	Corresponding Tricks
Silver-based dressings: effective antimicrobial action but cytotoxic to keratinocytes, fibroblasts, MSCs; destabilize EV membranes; generate ROS	Short-term use during acute infection; transition to cell-friendly dressings; non-adherent barriers (e.g., silicone) to separate silver from therapeutic cells; ECM or hydrogel scaffolds for protection
Iodine preparations: reduce microbial load and biofilms but impair proliferation and migration of MSCs and host cells	Careful dosing and duration; staged use with rapid switch to supportive carriers; integration with less cytotoxic antimicrobials
Brilliant green (zelyonka): historically popular antiseptic, but strongly cytotoxic to keratinocytes, fibroblasts, MSCs, and destabilizes EVs	Limit to very short-term use, if at all, for surface disinfection; avoid concurrent application with regenerative therapies; transition rapidly to biocompatible dressings
Residual infection/biofilm: hypoxic, acidic niches with proteases degrade EV cargo; bacterial EVs mimic host vesicles and spread pro-inflammatory signals	Quorum-sensing inhibitors, bacteriophages, targeted antibiotics; staged antimicrobial + regenerative protocols; biofilm-disrupting adjuncts (enzymes, peptides)

Broad antiseptics (chlorhexidine, hydrogen peroxide, polyhexanide): disrupt lipid membranes, induce oxidative stress, leading to collateral damage to MSCs/EVs	Limit exposure to early wound cleansing; follow with regenerative-compatible dressings; buffer solutions to reduce cytotoxic carryover
Antibiotics (systemic/topical): some impair mitochondrial function or shift microbiome balance, which leads to reduced MSC viability and altered EV signaling	Rational antibiotic selection; monitoring host-microbiome balance; exploring microbiome-supportive adjuncts to preserve regenerative cross-talk

Supplementary Table S3. Trap-Trick Capsule: Dosing and Delivery Pitfalls. Key Paradox: Dosing Paradox - Higher doses increase washout, stress, and clearance, leading to paradoxical loss of efficacy

Traps	Corresponding Tricks
Wound exudation and shear forces wash away topically applied MSCs/EVs	Encapsulation in hydrogels, fibrin glue, or ECM scaffolds to anchor regenerative products
Intralesional injection leads to hypoxia, nutrient deprivation, and loss of anchorage, which cause rapid MSC death	Biomaterials providing structural support and oxygenation; preconditioning cells for hypoxic tolerance
Exosomes diffuse freely, are adsorbed into dressings, or undergo rapid endocytic clearance	Slow-release carriers (hydrogels, patches, 3D-printed scaffolds); surface modification with targeting ligands
Transient exposure to cues is insufficient to reprogram the chronic wound trajectory	Repeated dosing schedules; sustained-release systems maintaining therapeutic presence
Dosing paradox: high bolus doses increase convective washout, metabolic stress, DAMP release, immune clearance, and receptor desensitization, which leads to paradoxical loss of efficacy	Fractionated, niche-aware dosing; smaller repeated applications; microvolume delivery formats; protective carriers; MSC preconditioning and EV stabilization
Stem Cell Bathing: topical irrigation with MSC suspensions or conditioned medium causes washout, receptor desensitization, apoptosis in unattached cells, DAMP release, and vesicle degradation	Redesign bathing as a niche-aware strategy: embed in hydrogels, ECM, or bioadhesive coatings; use fractionated irrigation instead of a single bolus; add protective agents (antioxidants)

Supplementary Table S4. Trap-Trick Encapsule: Fibrosis and Scarring Bias. Key Paradox: Scarring Paradox - MSCs/EVs may suppress or reinforce fibrosis depending on niche context

Traps	Corresponding Tricks
Wounds default toward fibrosis and scarring to restore the barrier rapidly	Frame therapies to modulate, not bypass, the fibrotic program
TGF-β, CTGF, YAP/TAZ signaling drive myofibroblast activation and ECM overproduction	Anti-fibrotic adjuvants (quercetin, losartan, verteporfin); senolytics targeting senescent fibroblasts
MSCs/EVs show dual roles: regenerative in permissive niches but pro-fibrotic in dysregulated ones. EVs from latent viruses, pathogens, and	Precondition MSCs, bias EV cargo, or pair with anti-fibrotic co-therapies to tilt the balance toward regeneration.

fungi sustain scarring and keloid formation (Scarring Paradox)	Engineer MSCs/EVs to filter or neutralize vexosomes
Burns and chronic wounds: closure achieved, but at the cost of rigid, hypertrophic scars	Biophysical modulation: soft hydrogels, aligned nanofibers, and mechanical conditioning of the niche

Supplementary Table S5. Trap-Trick Capsule: Immune Clearance and Neuroimmune Crosstalk. Key Paradox: Immune Double-Edged Sword - The same pathways that enable healing also accelerate therapeutic loss

Traps	Corresponding Tricks
Immune Double-Edged Sword: clearance by neutrophils, macrophages, and complement reduces therapeutic persistence	Preconditioning to lower DAMP release; engineering MSCs/EVs for reduced immunogenicity; shielding with biomaterials or membrane camouflaging
EV opsonization and trafficking to the liver/spleen leads to limited local persistence	Targeting ligands, controlled-release carriers, site-directed delivery
Immunosuppressive effects of MSCs/EVs may create tumor-supportive microenvironments	Careful patient selection, oncological screening, limited dosing; engineering secretome away from pro-angiogenic cargo
Risk of receptor over-suppression or immune imbalance	Dynamic dosing schedules; pairing with immunomodulatory adjuvants to maintain balance
Dysregulated neuroimmune crosstalk (CGRP, Substance P, vagal pathways) mis-educates immune cells toward hyper-clearance, chronic inflammation, fibrosis, and CNS micro-scarring (Neuroimmune Scarring)	Neuromodulatory adjuncts (vagus nerve stimulation, neuropeptide interventions); circadian-timed delivery of MSCs/EVs

Supplementary Table S6. Trap-Trick Capsule: Safety, Regulatory, and Economic Concerns

Traps	Corresponding Tricks
Regulatory ambiguity; variable classification (drug vs. ATMP vs. biologic); lack of standardized potency assays	Early dialog with FDA/EMA; harmonization of potency assays; standardized EV guidelines (MISEV)
GMP bottlenecks, batch variability, poor reproducibility	Bioreactor scaling, closed-system manufacturing, validated potency markers
High production cost; dependence on cold-chain storage	Allogeneic off-the-shelf sources; immortalized MSC lines; lyophilized or engineered EVs for ambient stability
Ethical gray zones; unregulated clinics; erosion of trust	Transparent communication, patient education, registries, enforcement of standards

Supplementary Table S7. Trap-Trick Capsule: Burns

Traps	Corresponding Tricks
Necrosis, vascular thrombosis, high inflammation lead to a hostile microenvironment for MSCs/EVs	Encapsulation in hydrogels, collagen, or ECM scaffolds to buffer stress and enhance survival
Natural bias toward fibrosis leads to hypertrophic scarring and contractures (Scarring Paradox)	Anti-fibrotic adjuvants (losartan, quercetin, verteporfin); biomaterial cues (aligned nanofibers, soft scaffolds)

Inconsistent clinical outcomes (accelerated closure but poor functional quality)	Integrative strategies combining microbial control, regenerative payloads, and scar-modulating adjuncts

Supplementary Table S8. Trap-Trick Capsule: Chronic Wounds. Key Paradoxes: Dosing Paradox + Reverse Scarring Paradox - Higher doses worsen clearance; failure to mount adequate fibrotic closure leads to persistent open wounds

Traps	Corresponding Tricks
Persistent biofilms sustain proteolysis, oxidative stress, and degrade exosomal cargo	Quorum-sensing inhibitors; bacteriophages; enzymatic biofilm disruptors
Immune mis-education leads to macrophages and neutrophils locked in pro-inflammatory states	MSC/EV-derived microRNAs to reprogram macrophages; adjunct neuromodulation (vagal stimulation)
Dosing Paradox: higher doses increase washout, stress, and clearance	Fractionated, niche-aware dosing; microvolume delivery formats; encapsulation in hydrogels or microneedles
Poor retention due to exudate and ischemia	ECM-based scaffolds, oxygenating biomaterials, angiogenic carriers
Scarring Paradox (reverse): failure to mount closure leads to a persistent open wound	Controlled pro-fibrotic cues (temporary ECM scaffolds, targeted TGF-β modulation) paired with regenerative therapies

Supplementary Table S9. Trap-Trick Capsule: Grafting Strategies. Key Paradox: Scarring Paradox - MSCs/EVs accelerate closure but may reinforce fibrosis in dysregulated niches

Traps	Corresponding Tricks
Ischemia beneath the graft leads to necrosis of graft tissue and transplanted MSCs	Pro-angiogenic scaffolds (VEGF, oxygen-releasing biomaterials); pre-vascularized graft constructs
Inflammation and neutrophil proteolysis degrade graft and exosomal cargo	MSC/EV-loaded scaffolds to buffer early stress; antioxidant adjuvants
Integration mismatch leads to disorganized ECM, poor dermal-epidermal junctions	Mechanical conditioning (aligned nanofibers, elastic supports); scaffold design guiding ECM deposition
Scarring Paradox: MSCs/EVs accelerate closure but reinforce fibrosis if the niche is dysregulated	Timing of application (post-stabilization), pairing with anti-fibrotic adjuvants

Supplementary Table S10. Trap-Trick Capsule: Oral Ulcers. Key Paradoxes: Dosing Paradox + Microbiota-Exosome Axis - High doses accelerate clearance; microbial EVs compete with therapeutic signals

Traps	Corresponding Tricks
Rapid washout by saliva leads to poor retention	Mucoadhesive carriers (chitosan, hyaluronic acid, alginate)
Microbiota-Exosome Axis in oral ulcers: dysbiotic bacterial/fungal EVs and latent viral EVs compete with therapeutic MSC/EV signals, blunting immune reprogramming and prolonging inflammation	Probiotics or microbiome modulation to rebalance microbial EV output; engineered EVs resistant to microbial uptake; adjunct antivirals/antifungals to suppress hidden pathobiota; microbiome-stabilizing approaches

Mechanical shear (mastication, speech) disrupts applications	Dissolvable films, lozenges, oral patches with sustained release
Dosing Paradox: high-dose liquid suspensions accelerate clearance	Fractionated or sustained-release dosing in mucoadhesive carriers
Neuroimmune amplification (Substance P, pain signals) worsens clearance and inflammation	Pairing MSC/EV delivery with analgesics or neuroimmune modulators

Supplementary Table S11. Trap-Trick Capsule: Preconditioning and Biomaterial Carriers. Key Paradox: Preconditioning Paradox - Stressors destructive in vivo become constructive preparation tools in vitro

Traps	Corresponding Tricks
MSC fragility under hypoxia, ROS, and immune attack	Preconditioning (hypoxia, cytokine priming, oxidative stress training) to enhance resilience
EV washout and rapid clearance	Encapsulation in hydrogels, ECM scaffolds, or 3D-printed carriers for gradual release
Higher doses worsen washout and clearance (Dosing Paradox)	Fractionated microvolume delivery (sprays, microneedles) combined with carriers

Supplementary Table S12. Trap-Trick Capsule: Exosome Engineering and Pathology Filtration. Key Paradox: Exosome Paradox - The same property that makes EVs therapeutic also makes them vehicles of pathology

Traps	Corresponding Tricks
Double agency: EVs mirror parent cell state and may transmit pathological signals (senescence, fibrosis, microbial competition)	Filtration and sorting (size exclusion, immunoaffinity, microfluidics) to enrich regenerative vesicles and exclude pathological subtypes
Lack of targeting and rapid off-site clearance	Engineering EVs with targeting ligands or membrane coatings for improved homing
Inconsistent therapeutic cargo due to variable MSC culture conditions	Preconditioning or genetic modification of MSCs to package specific therapeutic microRNAs, proteins, or ligands

Supplementary Table S13. Trap-Trick Capsule: Anti-Fibrotic and Senolytic Combinations. Key Paradox: Scarring Paradox - MSCs/EVs may suppress or reinforce fibrosis depending on niche context.

Traps	Corresponding Tricks
Scarring Paradox: MSCs/EVs may suppress or reinforce fibrosis depending on niche context	Pair MSCs/EVs with anti-fibrotic agents (pirfenidone, nintedanib, quercetin) to bias signaling toward regeneration
Senescent cell populations secrete pro-fibrotic factors (SASP) that override regenerative cues	Senolytic drugs or senolytic-loaded nanoparticles to selectively clear senescent cells
EVs reprogrammed by fibrotic niches to propagate scar signals	Engineer vesicles to carry anti-fibrotic microRNAs or deliver them within anti-fibrotic scaffolds

Supplementary Table S14. Trap-Trick Capsule: Patient Stratification and Personalization. Key Paradox: Stratification Paradox - The more heterogeneous the cohort, the less effective uniform therapy appears.

Traps	Corresponding Tricks
Failed RCTs due to patient heterogeneity dilute therapeutic effects	Stratify patients by biomarkers (cytokines, EV cargo, wound metabolites) or wound type
Uniform "one-size-fits-all" therapy mismatched to diverse niches	Personalize dosing, timing, and adjuncts according to trap profiles
Inability to scale individualized assessments in clinical practice	Use digital tools (AI imaging, machine learning, multiomic integration) to automate stratification

Supplementary Table S15. Trap-Trick Capsule: Bioelectric Reset

Traps	Corresponding Tricks
Electrical field collapse and disorganization lead to incoherent electrotaxis, impaired closure; transplanted MSCs/EVs lack spatial guidance	Bioelectric reset via external stimulation or conductive biomaterials to restore polarity; closed-loop systems for precision control
External fields are spatially diffuse and temporally static	Bioelectric-optogenetic hybrids for spatiotemporal precision in regenerative programming

Supplementary Table S16. Trap-Trick Capsule: Herbal Paradox. Key Paradox: Herbal Paradox - Natural compounds dismissed as low-tech intervene at precise systemic choke points

Traps	Corresponding Tricks
Herbal compounds dismissed as anecdotal, inconsistent, or low-tech	Reframed as programmable regulators of EV cargo, immune tone, and microbiota interactions. Systematic extraction, standardization, and integration into modern protocols
Untapped capacity to modulate MSC/EV secretomes and microbial EVs	Use in MSC culture/preconditioning; co-formulation with EVs; systemic delivery to recalibrate microbiota.
Poor solubility and penetration of many phytochemicals. Cross-kingdom vesicles from microbes or plants compete with regenerative cues	Encapsulation in nanoparticles, embedding in hydrogels, or integration into MSC/EV scaffolds for controlled release. Harness or engineer cross-kingdom EVs to bias immunity and niche tone toward regeneration

Supplementary Table S17. Trap-Trick Capsule: Circadian Synchronization. Key Paradox: Circadian Paradox - Rhythms that undermine therapy when ignored amplify it when harnessed

Traps	Corresponding Tricks
Temporal blindness: ignoring circadian oscillations in immunity, perfusion, and proliferation	Align MSC/EV delivery with circadian repair windows using chronotherapy protocols
Circadian disruption in chronic wounds, stress, or diabetes	Reset rhythms with melatonin, vagal stimulation, or cortisol modulators
Uniform dosing in trials blunts the potential benefit	Patient-specific chronotherapy guided by digital chronobiology tools and wearables

Supplementary Table S18. Trap-Trick Capsule: Digital Twins and Predictive Simulation. Key Paradox: Predictive Simulation Paradox - The Complexity that makes wounds unpredictable becomes a source of precision when modeled

Traps	Corresponding Tricks
Retrospective evaluation obscures dynamic traps	Patient-specific digital twins that simulate wound trajectories in real time
Complexity and heterogeneity make outcomes unpredictable	Predictive models that integrate immune, microbial, EV, and electrical data into dynamic simulations
One-size-fits-all treatment strategies fail across diverse wounds	AI-guided personalization of MSC/EV timing, dosing, and combination therapies
Current drug discovery is slow, narrow, and trial-and-error	AI-guided discovery of new compounds that modulate EV cargo, fibroblast signaling, or metabolism

Supplementary Table S19. Trap-Trick Capsule: Erythrocyte Plasticity. Key Paradox: Erythrocyte Plasticity Paradox - Cells designed for maximal specialization may conceal maximal plasticity

Traps	Corresponding Tricks
Dogma of terminal erythrocytes as inert oxygen carriers	Recognition of erythrocyte plasticity (ACA-mediated) as a latent regenerative resource
Assumption that the absence of nuclei precludes regenerative function	Surface glycoproteins and vesicle trafficking as potential levers of programming
Circulating red cells overlooked in regenerative biology	Erythrocytes reframed as systemic reservoirs and programmable delivery vehicles

Supplementary Table S20. Trap-Trick Capsule: Mechanobiology and Metabolic Horizons. Key Paradoxes: Mechanobiology + Metabolic Paradoxes - Destructive forces and metabolic rigidity become therapeutic levers when controlled

Traps	Corresponding Tricks
Abnormal stiffness, strain, or shear bias fibroblasts toward fibrosis	Low-intensity ultrasound, acoustic fields, and nanostructured scaffolds to guide constructive alignment
Chronic wounds locked in glycolytic bias and mitochondrial dysfunction	Metabolic reprogramming with NAD^+ boosters, ketone esters, amino acid therapies to restore flexibility and redox balance

Supplementary Table S21. Trap-Trick Capsule: DMSO. Key Paradox: Vector Paradox - DMSO can carry toxins deep into tissue or be harnessed to deliver therapeutic agents

Traps	Corresponding Tricks
Cryoprotectant at high concentrations leads to cytotoxicity; MSC/EV damage if inadequately washed out	Careful dose titration; optimized cryorecovery protocols; reframing DMSO as a niche modulator and vector, worthy of systematic evaluation as an adjunct in wound healing.
Anecdotal, unstandardized clinical use leads to inconsistent outcomes, regulatory skepticism	Structured clinical trials; formal safety and efficacy evaluation
Tissue irritation with unregulated topical use	Controlled formulations with protective carriers
The vector property may carry contaminants or irritants deep into tissue	Vector property can be harnessed to deliver therapeutic molecules or adjuncts (e.g., antioxidants, herbal bioactives) deep into wound tissue

Supplementary Table S22. Comparative clinical and translational attributes of mesenchymal stem cell (MSC) and exosome-based therapies in wound healing. The table highlights mechanistic distinctions, safety considerations, manufacturing feasibility, regulatory pathways, and therapeutic contexts. Exosome-based products currently demonstrate greater near-term potential for scalable and standardized clinical translation, whereas MSCs remain essential for complex tissue remodeling and as cellular biofactories for regenerative vesicle production.

Feature / Parameter	Mesenchymal Stem Cells (MSCs)	MSC-Derived Exosomes (EVs)	Comparative Note
Therapeutic Mechanism	Paracrine + cell–cell signaling; secretion of trophic and immunomodulatory factors	Cell-free delivery of MSC-derived regulatory cargo (miRNAs, cytokines, proteins)	Exosomes reproduce much of the MSC paracrine activity
Safety Profile	Low, but not negligible, risk of immune rejection or ectopic differentiation	Non-replicative; minimal immunogenicity	Exosomes safer for repeated topical use
Manufacturing and Storage	GMP expansion required; donor variability; cryostorage at −80 °C	Scalable purification; lyophilizable; ambient stability under development	Exosomes easier to standardize and distribute
Regulatory Pathway	Classified as ATMP/biologic (complex oversight)	Closer to biologic/drug category (clearer regulatory path)	Exosomes more straightforward for approval
Clinical Evidence	Moderate evidence from early RCTs in burns and ulcers; variable outcomes	A growing number of trials with consistent safety and efficacy signals	Exosomes showing more reproducible benefit
Best-fit Indications	Deep or complex wounds, ischemic defects, reconstructive grafts	Chronic or superficial wounds requiring repeated, standardized dosing	Often complementary
Current Limitation	Immune clearance, poor retention, high cost	Heterogeneity, potency assays, scalability of isolation	Both need standardization
Overall Translational Potential	High for specialized or autologous use	Higher for near-term clinical translation and broad scalability	

Supplementary Table S23. Regulatory and Safety Overview of Key Modalities Referenced in the Manuscript. Purpose: To summarize regulatory status, evidence maturity, and known safety considerations of selected agents and interventions mentioned in the perspective review. The table is provided for transparency and does not imply clinical endorsement.

Modality / Intervention	Current Regulatory Status	Known Adverse Effects	Contraindications / Precautions	Notes
Silver / Iodine Dressings	CE-marked, FDA-cleared for wound care (various formulations)	Local irritation, delayed epithelialization at high doses	Hypersensitivity; avoid systemic absorption in large wounds	Safe when used within approved indications
HOCL (hypochlorous acid)	FDA-cleared and CE-marked wound irrigant	Mild stinging; rare irritation	None specific; avoid ingestion	Low-toxicity antimicrobial
Medical-Grade Honey	CE-marked, FDA-cleared (various brands)	Allergy (rare), transient pain	Known allergy to honey/pollen	Antimicrobial and osmotic

				activity; broad clinical use
Phage Therapy	Experimental use in EU and US	Infusion reactions, endotoxin release	Severe immunosuppression (relative)	Requires GMP preparation;
Senolytics (quercetin, dasatinib, verteporfin)	Experimental; under clinical trials	Cytopenia, hepatic enzyme elevation, phototoxicity	Pregnancy, liver dysfunction	Preclinical antifibrotic and rejuvenative evidence only
Vagal Stimulation	FDA-approved for epilepsy, depression; investigational in wound healing	Voice change, bradycardia, cough	Cardiac conduction disorders	Noninvasive approaches (auricular) under evaluation
MSC and MSC-EV Products	Regulated as ATMPs / biologicals (EMA, FDA)	Immune reactions, infusion site pain	Active malignancy, infection	Approved for limited indications; wound use investigational
Herbal Bioactives (e.g., Curcumin, Plantain)	Dietary supplement category; not regulated as drugs	GI upset, allergic rash	Herbal–drug interactions	Use limited to supportive care; variable purity
Electrical Stimulation Devices	CE-marked and FDA-cleared for chronic wounds	Skin irritation, burns (rare)	Pacemaker, metal implants	Protocol standardization needed
DMSO **(**as vector/carrier**)**	Approved as Rx (interstitial cystitis, organ preservation)	Garlic odor, local irritation, headache	Sulfa allergy, hepatic impairment	Off-label for wound use; limited human data

Supplementary Table S24. Clinical Landscape of MSC and MSC-Derived EV Trials in Wound Healing (Scoping Summary) [95,160,274–287]. Purpose: To anchor the traps–paradoxes–tricks framework in representative clinical evidence. Note: Values reflect key published trials; not a systematic extraction. PRP- Platelet-Rich Plasma.

**Condition**	**Therapy**	**Study Design**	**Main Outcome**	**Adverse Events**	**Evidence Level**
Diabetic Foot Ulcer (DFU)	MSCs, EVs	Clinical and preclinical	Improved healing rate for small ulcers	No significant SAEs for small ulcers	A<br>C (EVs)
Venous Leg Ulcer (VLU)	MSCs and PRP	Case Report, Meta-analyses	Completely healed within two months	Mild local pain	B
Burn Wounds / Graft Take	MSCs	Meta-analyses	Faster epithelialization; improved graft adherence, faster healing, suppresses inflammation	Reduction in SAE	C-B
Hypertrophic and Keloid Scars	MSCs, EVs	Case reports, Meta-analyses	Reduction in thickness, color hue, and volume	None	C-B

Supplementary Horizon Table S25. Emerging Frontiers in Regenerative Wound Therapy. Purpose: To summarize the conceptual and preclinical status of themes discussed in Section 5 (Bioelectric Reset, Herbal Paradox, Erythrocyte Plasticity, Vector Paradox), and related constructs in Section 6.

Concept	Rationale / Mechanistic Focus	Evidence Status	Caution
Bioelectric Reset (5.1)	Restoration of wound polarity and electrical guidance through exogenous stimulation or conductive scaffolds	Early animal + pilot human trials	Safety and parameter standardization (current density - burn risk, pacemaker interference) pending
Herbal Paradox (5.2)	Plant-derived bioactives showing both regenerative and inhibitory effects depending on dose/context	Mainly in vitro / rodent	Variable purity, drug interactions; avoid clinical extrapolation
Vector Paradox (5.3)	Solvent carriers (DMSO, etc.) modifying membrane permeability and signaling, enhancing or impairing regenerative payloads	Mechanistic studies only	Off-label use risks; requires dose and toxicology validation
Circadian Synchronization (5.4)	Aligning therapy timing with circadian immune-repair windows; chronotherapy of wound care	Preclinical and observational data; pilot human evidence	Needs controlled trials and device synchronization protocols
Erythrocyte Plasticity (5.5)	RBC deformability and EV release in oxygenation and inflammation	Supported by biophysical and clinical correlations	Translational applications remain conceptual
Digital Twins and AI Modeling	Data-driven simulation of patient-specific wound dynamics and therapy response	Concept validated in other domains; early feasibility in wound care	Needs large datasets, interoperability, privacy safeguards
Mechanobiology / Micro-Tensional Cues	Substrate stiffness and mechanical load influence cell fate, EV release, and matrix remodeling	Preclinical and organ-on-chip data	Translation and safety validation required
Vexosomes (6.2)	Pathological EVs propagating microbial and fibrotic signals within the Triad-Exosome Axis	Mechanism supported; framework conceptual	Requires biomarker validation and quantitative criteria

Note: Representative references are cited within the corresponding subsections of the main text.

Supplementary Table S26. Integrative and Emerging Adjuncts Potentially Influencing Wound Repair - Hypothesis-Generating Evidence. Purpose: To collate integrative or non-standard modalities that have reported favorable outcomes in limited or practice-based contexts. Inclusion does not imply endorsement or clinical validation; the table identifies hybrid or ecosystem-level interventions meriting systematic investigation.

Modality / Concept	Proposed Mechanism	Observations	Evidence Source / Type	Research Need

Polyherbal or phyto-nutrient combinations	Antioxidant, antimicrobial, pro-angiogenic phytochemicals; microbiome modulation	High closure rates and reduced inflammation	Case reports, observational integrative clinic data	Controlled RCTs with standardized extracts and dosing
Probiotic or microbiome reset therapy	Restoration of commensal dominance; attenuation of dysbiosis-driven inflammation	Faster granulation, odor reduction	Pilot human studies; in vitro biofilm assays	Mechanistic and microbiota-sequencing validation
Photobiomodulation / frequency-based stimulation	Resonant or low-level light fields restoring cellular redox balance and mitochondrial coupling	Accelerated closure, reduced pain and exudate	Small uncontrolled cohorts; mechanistic cell data	Parameter optimization; sham-controlled trials with dosimetry reporting
Scalar or bio-resonant field modulation	Hypothesized coherence effects on cell signaling and water structuring	Anecdotal reports of rapid tissue normalization	Practice-based experience; no peer-reviewed data	Foundational biophysical studies; reproducibility testing
Hybrid detox-nutritional protocols	Synergistic dietary, supplements, and phytotherapeutic regimens aimed at reducing systemic inflammatory and toxic load (trace metal, viral, bacterial, or parasitic) and restoring metabolic homeostasis, often complemented by enteric binders that limit reabsorption of microbial and metal metabolites	Systemic symptom improvement, improved healing	Case series, practice-based observations	Controlled metabolic, toxicologic, and microbiome studies with standardized hybrid protocols

Note: All listed interventions remain experimental and are discussed for hypothesis generation only. Controlled pre-clinical and clinical research is required before therapeutic application.

Supplementary Table S27. Reference Ranges, simplified. Note: Measuring "wound potential" in mV/mm is not a current clinical practice. It is a research technique. Diagnosis and prognosis of chronic wounds rely on standardized clinical assessments (perfusion, infection, size, etiology). EF - Electric Field.

Wound State	Electric Field Strength	Interpretation and Context
Acute wounds (Experimental Models)	~20–200 mV/mm	Normal healing response. Observed range in lab models. The field is a key guidance cue for epithelial cell migration, neutrophil chemotaxis, and stem cell orientation. It is considered essential for coordinated healing.
Chronic wounds	<10 mV/mm. Weak, Disorganized, or Absent	The failure to generate or sustain a physiological EF is hypothesized to be a key factor in the failure to heal. The specific threshold of "<10 mV/mm" is a plausible research hypothesis for a non-healing

		state, but it is not a universally accepted clinical cutoff. Impaired healing, poor prognosis
Healing trajectory (Predictive Target)	Sustained field >15 mV/mm	Predicts wound closure. Restoring and maintaining a field within the physiological range should predict a return to healing. The exact value is a therapeutic target for bioelectric devices, not a standard prognostic test.

Supplementary Table S28. Clinical application of Electrical Stimulation (ES) for wound healing.

Modality / Device	Key Clinical Parameters	Safety and Dosing Constraints
HVPC (High-voltage Pulsed Current)	Effective for DFU, PU, and VLU. Promotes perfusion, reduces biofilm, and directs cell migration.	Dosing: 30-60 min/session, 3-5x/week. Use sensory-level (tingling) or motor-level (visible contraction) amplitude. Safety: 1) Contraindicated in patients with pacemakers/ICDs. 2) Avoid placement that crosses the chest. 3) Do not use over the carotid sinus.
Biphasic Pulsed Current	Effective for inflammatory states (VLU). Can modulate inflammation and promote healing.	Dosing: 30-60 min/session, 3-5x/week. Sensory-level amplitude. Safety: Same as HVPC. Generally considered to have a lower risk of skin irritation than monophasic currents.
LIDC / Microcurrent	Efficacy is highly dose-dependent. Underpowered/under-dosed show no effect. Requires adequate session duration and current intensity.	Dosing: Critical: Sessions must be >30 minutes. The current must be sufficient to produce a biological effect. Safety: 1) Low risk, but the same general contraindications apply. 2) Ensure good electrode contact to prevent current hotspots.
Bioelectric Dressings	Emerging technology. Early clinical signals for improved perfusion and closure in pilot studies. Convenience of continuous, low-level delivery.	Dosing: Changed with standard dressing cycle (e.g., every 1-3 days). Safety: 1) Monitor for sensitivity to dressing components. 2) Contraindicated in patients with device sensitivity to the specific battery/metals used. 3) Not for use with MRIs.
TENS (for wound outcomes)	Not recommended for wound healing. Evidence is mixed and inconsistent. Its primary and validated purpose is analgesia.	Dosing: N/A for wound healing. Safety: Should not be relied upon as a primary wound healing modality. Use only for its intended purpose of pain control.

General Safety Constraints. The application of ES must be governed by strict safety protocols to prevent harm. Summary of Overarching Safety Principles: 1. Diagnosis First: ES is an adjuvant therapy, not a replacement for standard of care (debridement, offloading, compression, moisture balance, infection control). 2. Contraindications are Absolute: Never ignore contraindications related to implanted electronics, malignancy, or thrombosis. 3. Patient-Specific Application: Parameters (polarity, intensity) should be selected by a trained clinician based on wound assessment. 4. Informed Consent: Patients must be educated on the goals, expectations, and safety protocols of the therapy.

General Contraindications and Precautions

Placement Over Malignancy: Do not apply ES over a known or suspected malignant tissue site due to the theoretical risk of stimulating tumor growth.

Placement Over Thrombophlebitis: Avoid application directly over an area of active deep vein thrombosis (DVT) or thrombophlebitis.

Presence of Implanted Electronic Devices: Absolute contraindication. ES can interfere with the function of cardiac pacemakers, implantable cardioverter-defibrillators (ICDs), and spinal cord stimulators. The electrical current can be misinterpreted by the device, leading to life-threatening malfunction.

Over the Carotid Sinus / Neck Region: Avoid stimulation in this area due to the risk of acute hypotension or bradycardia.

Wound-Specific Precautions

Untreated Osteomyelitis: ES is not a treatment for bone infection. The underlying infection must be managed medically or surgically first.

Active Bleeding: Do not apply to a wound with active, uncontrolled hemorrhage.

Precautions with Ischemic Wounds: Use with caution in wounds with critical limb ischemia. While ES can improve perfusion, it should be part of a comprehensive vascular management plan. It is not a substitute for revascularization when indicated.

Application-Specific Safety

Electrode Placement: Electrodes should never be placed in a way that current would pass transversely across the chest or through the brain.

Skin Integrity under Electrodes: Inspect the skin under the electrodes frequently for signs of irritation, allergic reaction, or breakdown. Use appropriate conductive gel or self-adhesive electrodes to ensure even current distribution and prevent burns.

Patient Sensation: For sensory-level stimulation (like TENS or some HVPC protocols), the sensation should be strong but comfortable and never painful. Pain indicates the amplitude is too high or there is a problem with the electrode contact, which can lead to skin damage.

Electrical Safety: Use only medical-grade, FDA-cleared/CE-marked devices that are regularly inspected for safety. Frayed wires or damaged devices must not be used.

Dosing and Professional Oversight

Not a Home-Monotherapy: Especially for complex chronic wounds (DFU, VLU, PU), ES should be administered by or under the direct supervision of a trained clinician (e.g., physical therapist, wound care nurse) as part of a comprehensive wound care program that includes debridement, offloading, compression, and moisture balance.

Parameter Selection: The choice of waveform, polarity, pulse parameters, and treatment duration should be based on the wound's characteristics (e.g., using negative polarity for heavily infected wounds initially) and current clinical practice guidelines.

Supplementary Note S1. Prioritized Trial Agenda for Next-Generation MSC/EV-Based Wound Therapies. Disclaimer: This agenda outlines research hypotheses, candidate comparators, and mechanistic endpoints for future controlled trials. It is not intended as clinical guidance or practice direction.

1. Microenvironment-Aligned Sequencing Trials

Objective: Test whether sequential optimization (infection → metabolic state → MSC/EV dosing) outperforms direct bolus MSC/EV therapy.

Design Priorities:

- Population: Diabetic foot or venous leg ulcers with defined pH, lactate, $tcPO_2$ ranges.
- Arms:
    - MSC/EV alone
    - Antimicrobial optimization → MSC/EV
    - Antimicrobial + metabolic support → MSC/EV
- Endpoints: EV uptake, inflammation markers, microbial EV load, 12-week closure rate.

- Mechanistic correlative: EV cargo profiling, bioburden sequencing.

2. Fractionated vs. Bolus EV/MSCs Dosing

Objective: Determine whether fractionated microvolume dosing improves retention and efficacy vs. single bolus.

Design Priorities:

- Platforms: Microneedle array, sprayable film, hydrogel patch.
- Comparators:
    - Bolus free MSC/EV
    - Fractionated free MSC/EV
    - Fractionated hydrogel-embedded MSC/EV
- Endpoints: pain, VSS/POSAS, elasticity, 6-month scar quality.
- Mechanistic correlative: in vivo EV tracking, wound-potential gradients.

3. Senolytic + MSC/EV Combination Trials

Objective: Evaluate whether selective removal of senescent cells (SASP sources) enhances regenerative potency.

Design Priorities:

- Agents: Low-dose quercetin, fisetin, navitoclax (topical/adjunctive).
- Arms:
    - MSC/EV alone
    - Senolytic priming → MSC/EV
- Endpoints: fibrosis markers, VEGF/TGF-β ratios, collagen alignment.
- Mechanistic correlative: miR-29, senescence burden (p16, p21), mast-cell EV profile.

4. Chronotherapy of Regenerative Interventions

Objective: Assess whether aligning MSC/EV therapy with circadian immune-metabolic windows improves outcomes.

Design Priorities:

- Timing: morning vs. evening vs. personalized wearable-derived rhythm window.
- Adjuncts: melatonin, vagal stimulation, cortisol waveform correction.
- Endpoints: granulation rate, cytokine oscillation amplitude, wearable-derived perfusion metrics.
- Mechanistic correlative: chronobiome (rhythmic EV cargo), local tissue clocks.

5. Bioelectric Reset + MSC/EV Hybrid Trials

Objective: Test whether restoring wound polarity via microcurrent/HVPC augments MSC/EV guidance and engraftment.

Design Priorities:

- Arms:
    - MSC/EV alone
    - Bioelectric reset alone
    - Bioelectric reset → MSC/EV
- Endpoints: rate of epithelial gap closure, VSS, graft take (if applicable).
- Mechanistic correlative: spatial electrotaxis mapping, wound voltage gradients.

6. Digital Twin–Guided Regenerative Protocols

Objective: Evaluate the feasibility and precision of AI-guided wound digital twins for dosing/timing recommendations.

Design Priorities:

- Inputs: imaging, pH, lactate, bioburden, EV cargo, perfusion, circadian metrics.

- Output: predicted healing trajectories with vs. without intervention.
- Endpoints: prediction accuracy, therapy responsiveness, inter-patient variability.
- Mechanistic correlative: dynamic EV signatures, microbiome shifts.

7. Multi-Axis Regenerative Strategies (Triad-Exosome Axis)

Objective: Test hypotheses derived from the Mito-Mast-Microbiota–Exosome integration model.

Design Priorities:

- Interventions:
    - mitochondrial support (NAC, phospholipids),
    - mast-cell modulation (ketotifen, cromolyn),
    - microbiota correction (topical probiotics),
    - MSC/EV therapy.
- Endpoints: inflammation coherence index, microbial EV burden, scarring profile.
- Mechanistic correlative: EV–axis coupling signatures (miR-146a, tryptase, LPS-EV load).

Supplementary Table S29. Clinical Measurement Panels for Triad/Axis Assignment of wounds to specific axis states within the Triad-Exosome framework. Core Panel (Point-of-Care, 15-minute assessment). *Optional metabolic-detox panel relevant to hybrid detox–nutritional interventions (see Supplementary Table S5).

Analyte	Sample Type	Platform	Axis Contribution	Clinical Interpretation
pH	Wound fluid	pH strip/meter	Microbiome, Metabolic	Chronic wounds are typically alkaline favoring protease activity, biofilm, and bacterial growth. Acidic shift (via dressings/acids) promotes healing.
Lactate	Wound fluid	Lactate strip	Metabolic	Elevated lactate is normal/required early in healing (stimulates angiogenesis/collagen). Persistently high suggests ischemia. No strict "severe" cutoff; context-dependent.
* Heavy-metal trace panel (Zn, Cu, Pb, Hg)	Serum / urine	ICP-MS/ LC-MS	Metabolic	Detects systemic toxic metals that may delay healing. No established role as routine chronic wound biomarkers. Zn/Cu deficiency can impair healing; excess Pb/Hg rare/direct cause. Note: investigational
*Endotoxin (LAL or aptamer) before / after binder therapy	Plasma / stool	ELISA / aptame	Microbiome–Metabolic	Evaluates binder-mediated reduction in circulating LPS. Promising research area (metabolic endotoxemia → low-grade inflammation delaying healing), but not validated for routine monitoring/response bands. Binders experimental.
TEWL	Intact periwound	Evaporimeter	Immune, Mechanobiology	Barrier function indicator; high values suggest inflammation

Analyte	Sample Type	Platform	Axis Contribution	Clinical Interpretation
$tcPO_2$	Periwound skin	Clark electrode	Metabolic	Direct measure of tissue oxygenation; critical for healing prognosis
Bioburden/Biofilm	Wound surface	Visual score + ATP	Microbiome	Quantitative bacterial load;
Wound Potential	Wound-intact skin	Surface electrode	Bioelectric	Endogenous electric field strength; correlates with migration
CRP	Capillary blood	Lateral flow	Immune	Systemic inflammation marker
Pain VAS	Patient report	Visual analog scale	Neuroimmune	Neuropathic vs nociceptive patterns inform axis dysfunction

- **Basic Traffic Light Interpretation Schema**
  - Green (Proceed with regenerative therapy): Core panel ≥6/8 parameters in normal range;
  - Yellow (Optimize microenvironment first): Core panel 3–5/8 parameters abnormal; Consider sequential intervention
  - Red (Address dysfunction before cell therapy): Core panel ≥6/8 parameters abnormal; Requires aggressive intervention
- **EV Characterization Panel (Send-out, weekly/bi-weekly)**

Supplementary Table S30a. Specialized panel for extracellular vesicle analysis to assess regenerative capacity and pathological signatures.

Analyte	Sample Type	Platform	Axis Contribution	Clinical Interpretation
EV Concentration	Wound fluid	NTA (ZetaView/NanoSight)	All axes	Total vesicle burden; includes therapeutic and pathological EVs
miR-146a	EV-enriched RNA	RT-qPCR	Immune (anti-inflammatory)	NF-κB negative regulator; elevated in resolution
miR-21	EV-enriched RNA	RT-qPCR	Immune, Metabolic	Dual role – pro-healing at moderate levels (keratinocyte migration); high in chronic wound EVs may drive excess ECM/fibrosis. Pro-fibrotic when elevated; angiogenic at moderate levels
miR-29	EV-enriched RNA	RT-qPCR	Metabolic (collagen regulation)	Collagen suppressor; low in keloids/HTS, anti-fibrotic
EV-Endotoxin/LPS	EV surface	LAL assay or aptamer	Microbiome	Bacterial vesicle contamination indicator
IDO (EV-protein)	EV lysate	ELISA	Immune	Prevents overactive immune response that may damage new tissue; immunomodulatory
VEGF (EV-protein)	EV lysate	ELISA	Metabolic	Angiogenic capacity of therapeutic EVs

Supplementary Table S30b. Immune/Fibrosis Panel (Laboratory assessment)

Analyte	Sample Type	Platform	Axis Contribution	Clinical Interpretation
IL-6	Wound fluid/serum	ELISA/Luminex	Immune	Acute phase response; chronicity marker
IL-8	Wound fluid	ELISA/Luminex	Immune	Neutrophil chemotaxis; correlates with non-healing
TNF-α	Wound fluid	ELISA/Luminex	Immune	Pro-inflammatory; tissue destruction when elevated
TGF-β	Wound fluid	ELISA	Immune, Metabolic	Master fibrosis regulator; healing vs. scarring balance, signaling lost in chronic wounds
CTGF	Wound fluid	ELISA	Metabolic	Downstream TGF-β effector; fibroblast activation
MMP-9	Wound fluid	Zymography/ELISA	Immune, Mechanobiology	Matrix degradation; imbalance indicates chronicity
TIMP-1	Wound fluid	ELISA	Immune, Mechanobiology	MMP inhibitor; calculate MMP-9/TIMP-1 ratio
Mast Cell Tryptase	Wound fluid	ELISA	Immune (Triad node)	Mast cell degranulation marker; key triad component

Supplementary Table S30c. Microbiome Panel (Specialized assessment)

Analyte	Sample Type	Platform	Axis Contribution	Clinical Interpretation
16S rRNA profiling	Wound swab	NGS	Microbiome	Community structure; dysbiosis assessment
S. aureus load	Wound swab	qPCR (nuc gene)	Microbiome	Pathogen-specific quantification
P. aeruginosa load	Wound swab	qPCR (oprL gene)	Microbiome	Biofilm-former quantification
Quorum sensing (AHL)	Wound swab	Biosensor/LC-MS	Microbiome	Bacterial communication; biofilm coordination
Fungal load	Wound swab	qPCR (ITS region)	Microbiome	Often overlooked; important in diabetic wounds

Supplementary Table S30d. Mechanobiology Panel (Biomechanical assessment)

Analyte	Sample Type	Platform	Axis Contribution	Clinical Interpretation
Elastic Modulus	Wound edge	Cutometer	Mechanobiology	Tissue compliance; scarring risk assessment
Tissue Thickness	Wound bed	Ultrasound (high-freq)	Mechanobiology	Granulation tissue depth; healing progression
Shear Modulus	Periwound	Elastography	Mechanobiology	Tissue stiffness; contracture risk
Collagen Organization	Biopsy (optional)	SHG microscopy	Mechanobiology	Scar quality predictor

Supplementary Table S30e. Sampling Timeline and Decision Points

Time Point	Core Panel	EV Panel	Immune/Fibrosis	Microbiome	Mechanobiology	Clinical Decision
Baseline (Pre-debridement)	√	√	√	√	√	Establish triad state; identify dominant axis
Post-debridement Day 0	√		√	√		Assess immediate response; confirm microbial clearance
Week 1	√					Early trajectory; adjust antimicrobial strategy
Week 2	√	√	√	√		Mid-point assessment; consider MSC/EV if improving
Week 4	√	√	√	√	√	Major decision point; evaluate for advanced therapies
Week 8	√	√			√	Outcome assessment; scar prevention strategies
Week 12	√		√		√	Final evaluation; long-term management plan

Comprehensive Traffic Light Interpretation Schema

Green (Proceed with regenerative therapy)

- Core panel: ≥6/8 parameters in normal range
- pH 6.5–7.4, lactate <2, $tcPO_2$ >40, bioburden <10^5
- EV panel: miR-146a elevated, miR-29 normal/high, low endotoxin
- MMP-9/TIMP-1 ratio <3, tryptase <5

Yellow (Optimize microenvironment first)

- Core panel: 3–5/8 parameters abnormal
- pH <6.5 or >7.5, lactate 2–4, $tcPO_2$ 30–40
- Moderate bioburden, reduced wound potential
- Consider sequential intervention: antimicrobial → metabolic support → MSC/EV

Red (Address dysfunction before cell therapy)

- Core panel: ≥6/8 parameters abnormal
- pH extreme, lactate >4, $tcPO_2$ <30, bioburden >10^6
- High inflammatory markers, low miR-29, high endotoxin
- Requires aggressive debridement, infection control, systemic optimization

Notes on Implementation

Minimal viable panel: For resource-limited settings, prioritize: pH, lactate, $tcPO_2$, bioburden score, and wound potential

EV analysis standardization: Use MISEV for EV isolation; report particle-to-protein ratio

Sample collection: Wound fluid via occlusive dressing (Tegaderm) for 2–4 hours; avoid contamination with irrigation fluid

Biofilm scoring: Use validated clinical scores (e.g., Wound Biofilm Index) combined with objective measures

Integration with imaging: Consider thermography for inflammation mapping, fluorescence for bacterial distribution

Cost-effectiveness: Core panel ~$50–100; full assessment ~$500–1000, depending on EV analysis inclusion

Datasets and Assay Provenance

Public Wound Fluid Proteome/miRNA Resources

PRoteomics IDEntifications Database (PRIDE)

Access: www.ebi.ac.uk/pride/

Gene Expression Omnibus (GEO)

Access: www.ncbi.nlm.nih.gov/geo/

Extracellular Vesicles Atlas

Access: http://bioinfo.life.hust.edu.cn/EVAtlas

Wound Healing Society Repository

Access: www.woundheal.org/ (registration required)

Cochrane Wounds Group Specialised Register, and the Cochrane Central Register of Controlled Trials

Access: www.cochranelibrary.com

Validated measurement device: Dermacorder™: FDA-cleared for wound potential mapping

Bioelectric Potential Measurement

• Electrode specifications: Ag/AgCl, 2mm diameter
• Reference positioning: 2cm from the wound edge in intact skin
• Minimum sampling: 5 points around wound perimeter

Normal reference ranges:

• Acute wounds: 20–40 mV/mm (lateral field)
• Chronic wounds: <10 mV/mm
• Healing trajectory: Recovery to >15 mV/mm predicts closure

Clinical Use

- Baseline Assessment: Identify wound type and healing potential
- Monitoring: Track changes in field strength over time
- Intervention Planning: Consider electrical stimulation if the field remains <10 mV/mm
